# Analyzing protein conjugation reactions for antibody‐drug conjugate synthesis using polarized excitation emission matrix spectroscopy

**DOI:** 10.1002/bit.28229

**Published:** 2022-09-28

**Authors:** Ana L. de Faria e Silva, Alan G. Ryder

**Affiliations:** ^1^ Nanoscale BioPhotonics Laboratory, School of Chemistry National University of Ireland Galway Ireland

**Keywords:** antibody drug conjugate, conjugation, fluorescence, monoclonal antibody, polarized

## Abstract

Antibody‐drug conjugates (ADCs) are promising anticancer therapeutics, which offer important advantages compared to more classical therapies. There are a variety of ADC critical quality attributes (CQAs) such as the protein structure, aggregation, and drug‐to‐antibody ratio (DAR), which all impact on potency, stability, and toxicity. Production processes can destabilize antibodies via a variety of physical and chemical stresses, and or by increased aggregation after conjugation of hydrophobic drugs. Thus, a proper control strategy for handling, production, and storage is necessary to maintain CQA levels, which requires the use of in‐process quality measurements to first identify, then understand, and control the variables which adversely affect ADC CQAs during manufacturing. Here, we show how polarized excitation emission matrix (pEEM) spectroscopy, a sensitive, nondestructive, and potentially fast technique, can be used for rapidly assessing aggregation and DAR in a single measurement. pEEM provides several sources of information for protein analysis: Rayleigh scatter for identifying aggregate/particle formation and fluorescence emission to assess chemical and structural changes induced by attachment of a linker and/or a small molecule drug payload. Here, we used a nontoxic ADC mimic (monoclonal antibody with linker molecule) to demonstrate efficacy of the measurement method. Emission changes caused via light absorption by the attached linker, allowed us to predict DAR with good accuracy using fluorescence signal from the final purified products (6% relative error of prediction [REP]) and also from unpurified alkylation intermediates (11% REP). pEEM changes could also be correlated with size (hydrodynamic radius, *R*_h_) and aggregate content parameters obtained from dynamic light scattering and size exclusion chromatography (SEC). For the starting material and purified product samples, pEEM correlated better with *R*_h_ (*R* ^2^ = 0.99, 6% REP) than SEC determined aggregate content (18% REP). Combining both fluorescence and light scatter signals also enabled in‐process size quantification (6% REP). Overall, combining polarized measurements with EEM and Rayleigh scatter provides a single measurement, multi‐attribute test method for ADC manufacturing.

## Graphical abstract

Spectroscopic monitoring of antibody drug conjugate (ADC) synthesis can be challenging due to spectral overlap between reactants and products, and because of the need to simultaneously measure multiple process parameters. Here, the authors show how polarized excitation emission matrix (pEEM) can rapidly assess aggregation and quantify the drug to antibody ratio via a single parallel polarized EEM measurement. pEEM is thus a potential process analytical technology for real‐time reaction monitoring of ADC production.

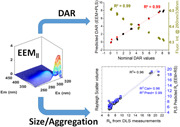

## 1 INTRODUCTION

Antibody‐drug conjugates (ADCs) couple the specificity of monoclonal antibodies (mAb) with the cell‐killing ability of cytotoxic agents. This is done to increase specificity toward tumor cells as well as improving pharmacokinetic profiles, and providing a wider therapeutic window (Wu & Senter, 2005). There are currently nine FDA approved ADCs with more than 80 molecules in clinical studies (Joubert et al., 2020). IgG1 is the most widely used antibody type for therapeutic purposes and also the most common protein found in ADCs in the market or in late‐stage clinical trials (Joubert et al., 2020). Chemical conjugation via lysine and cysteine are the most common ADC synthetic strategies (Joubert et al., 2020). Conjugation to the lysine amine group is widely used because it is relatively simple, often a single‐step reaction. However, it generates heterogeneous products because IgG has approximately 80 lysine‐derived amine groups (Mueller et al., 1988) of which approximately 10 are readily accessible for chemical modification. Cysteine conjugation is the major alternative, but because of the lack of free cysteine thiol groups in most proteins, the process usually first involves disulfide bond reduction under carefully controlled conditions to create sulfhydryl groups. These free thiols are then available for conjugation to reagents containing groups like maleimide. This route usually restricts the attachment sites to eight, leading to more homogeneous product mixtures compared to lysine conjugation (Jain et al., 2015), although site‐specific conjugation methods are now becoming available (Coumans et al., 2020).

Mishandling of proteins can lead to protein unfolding and aggregation, which can cause a loss in function and potentially cause immunogenicity issues for the patient (Sharma, 2007). In general, the most important critical quality attributes (CQAs) to be considered during protein modification are: homogeneity, purity, degree of conjugation, total protein concentration, and lot‐to‐lot variability of starting materials, intermediates, and final conjugated products. The CQAs ("Guidance for industry: Q8 [R2] pharmaceutical development, Guideline ICH Harmonized Tripartite," 2009) are the physical, chemical, or biological attributes of the drug substance/product known to impact product quality in terms of potency, pharmacokinetics, and toxicity (Alt et al., 2016; Raynal et al., 2014; Wagh et al., 2018). Protein and ADC analysis is technically demanding because of increased structural complexity compared to small molecules and the need to monitor both tertiary and quaternary structures. For ADC's, the problems are compounded by the fact that the small molecule payload also has to be characterized and that the structural changes caused by the payload (e.g., increased hydrophobicity) lead to products which are significantly more sensitive to aggregation.

Using intrinsic fluorescence measurements for protein structure and stability analysis is well established, because it involves minimal structural perturbation compared to the use of extrinsic labels, and is sufficiently sensitive (<10^–6^ M) (Quinn et al., 2015; Yadav et al., 2014). Simple (i.e., single point or single excitation measurements) intensity, lifetime, and anisotropy measurements can be implemented using relatively simple and inexpensive instrumentation and are widely used for studying processes like: aggregation (Ohadi et al., 2015), fibrillation (Bekard & Dunstan, 2009), unfolding (Vlasova & Saletsky, 2009), and binding (Lissi et al., 2013; Rawel et al., 2006; Soares et al., 2007; Zhang et al., 2008). However, most proteins are multifluorophore systems with photophysically active species present in close proximity (<10 nm) which interact via energy transfer and quenching. This generates complex emission which is better represented by 3D measurements like excitation emission matrix (EEM) (Warner et al., 1977) or total synchronous fluorescence spectroscopy (TSFS) (Patra & Mishra, 2002). Both have been used for multifluorophore mixture analysis for various applications (Bridgeman et al., 2011; Li et al., 2011, 2014; Ryan et al., 2010). By combining polarization with 3D EEM measurements, one can obtain extra information about changes in molecular size, local viscosity, and/or fluorophore mobility (Casamayou‐Boucau & Ryder, 2017; Groza, 2016; Groza et al., 2015).

Here, we investigated the use of polarized EEM (pEEM) to monitor an ADC reaction process with the key objectives being to: (1) measure variance of the mAb starting reaction materials, (2) monitor the course of the reaction and assess variance in the reaction intermediates, (3) predict final product drug to antibody ratio (DAR) during the alkylation reaction, and (4) measure variance and DAR of the final purified products. Most ADC analysis studies focus on characterizing final conjugate stability and DAR (Wakankar et al., 2011) with fewer looking at conjugation reaction monitoring. UV‐vis absorbance spectroscopy (Andris et al., 2018) was used to monitor DAR during conjugation of two different drug mimics to an engineered mAb. A RP‐HPLC and TOF mass spectrometry‐based method (Tang et al., 2017) was used to monitor DAR of a randomly conjugated lysine‐linked ADC. Our work presents a very different, multiattribute alternative for assessing aggregation changes during the reactions, measuring DAR during alkylation, and DAR in the final, partially purified product.

## 2 MATERIALS AND METHODS

### 2.1 Materials

mAb donated by Byondis was buffer exchanged before use (vide infra), the MC‐Val‐Cit‐PAB‐OH linker was purchased from Tokyo Chemical Industry, and TCEP hydrochloride, n‐acetylcysteine, and reagents for buffer preparation (NaH_2_PO_4_, Na_2_HPO_4*_7H_2_O and disodium EDTA dehydrate) were purchased from Sigma‐Aldrich. HPLC grade water (Fisher chemicals) was used for all solutions which were membrane filtered (0.10 μm) using Captiva filters before use. A single 11.0 ml mAb aliquot (see Supplemental Information, for more details) was buffer exchanged to remove the formulation buffer to facilitate synthesis. The mAb in reaction buffer was then aliquoted into smaller vials (5.0 ml LoBind tubes) suitable for single experiments, refrozen, and stored at –70°C until required. This reduces and controls the number of freeze‐thaw cycles ensuring that all samples in an experimental campaign have the same number of cycles. Absorbance spectroscopy was used to check the final concentration of mAb in PBS/EDTA and fluorescence measurements and dynamic light scattering (DLS) were also carried out (data not shown). Ellman's test (Supporting Information: Figure S1, Table S1) was used to verify the number of free thiol sites under the different conditions employed.

### 2.2 Linker and conjugation

A big challenge when studying ADCs is the often very high potency of the payload drug requiring the use of strict safety protocols and controlled environments. Thus, the safest alternative for preliminary studies particularly for analytical method development is to use a nontoxic model with payload molecules that mimic the structure/behavior of the real drug‐linker which facilitates the safe study of all the key steps from starting material preparation to final purification of conjugated products. The drug mimic used here was selected to be similar (e.g., solubility, absorptivity) to common drug‐linkers used in marketed ADCs but avoided toxicity issues. We used a molecule composed of valine, citruline, PAB‐OH, with an active maleimide terminal group, which is a common commercial ADC linker (Joubert et al., 2020). We selected a nonfluorescent small molecule because we wanted to minimize interference, and only measure the changes in protein fluorescence (Supporting Information: Figures S2 and S3).

Conjugates were prepared by partial reduction of IgG disulfide bonds with TCEP. HCl for 2 h followed by alkylation with an excess of the “drug” linker for 2 h, at 20°C. The two‐step reactions were conducted in 1 × 1 cm path length quartz cuvettes with slow stirring (using a flea magnetic follower in the cuvette with the sample holder stirrer) in the spectrometer (Supporting Information: Figure S3). A total of 24 reactions were performed, using 8 reducing agent TCEP concentrations from 0 to 50 molar excess (0, 1.25, 2.50, 5.0, 7.5, 10, 25, and 50) in triplicate. For all reactions, the same amount of “drug” linker (20‐fold excess compared to mAb concentration) and NAC quencher (12‐fold excess with respect to linker concentration) was used. The reaction mixtures were measured by absorbance and pEEM spectroscopy at 45‐minute intervals during the reaction (designated Red1‐/2‐/3‐IgG, Alk1‐/2‐/3‐/4‐IgG). The starting material (IgG‐SM), reduced intermediate (Red3‐IgG), unpurified final reaction mixture (Alk4‐IgG), and partially purified final products (Pur‐ADC) were also analyzed by DLS to look for aggregate formation (see Supporting Information: Tables S2 and S3). Because DLS, absorbance, and fluorescence measurements were made on the same cuvette sample we have confidence that all data relates to the exact same sample in terms of chemical and physical composition. Unpurified reaction mixtures were transferred to Eppendorf tubes and then stored at –70°C until purification was undertaken, several days post reaction. A simple filtration‐based purification (see Supporting Information) was implemented to remove unreacted small molecules (<10 kDa) and these partially purified samples (Pur‐ADC) were characterized by absorbance and fluorescence spectroscopy, DLS, SEC, and sodium dodecyl sulfate‐polyacrylamide gel electrophoresis (SDS‐PAGE).

### 2.3 Instrumentation and data collection

Absorbance spectra were collected using a Cary 60 spectrometer (Agilent) from the same cuvettes used for fluorescence measurements. Polarized EEM spectra were collected from 1 × 1 cm quartz cuvettes (Lightpath Optical) using a Cary Eclipse fluorescence spectrophotometer (Agilent) fitted with wire grid polarizers (Casamayou‐Boucau & Ryder, 2017) and a temperature controlled multicell holder. pEEM spectra were collected over an excitation range of *λ*_ex_ = 250–320 nm and λ_e__m_ = 290–450 nm emission range (2 nm increments in each case) with 10 nm excitation/emission slit widths and a scan rate of 1200 nm/min. Samples were measured under the four different polarization configurations, as previously described (de Faria e Silva et al., 2020a, 2020b), but only the parallel polarized EEM (EEM_||_) was used here because it is more sensitive to changes induced by conjugation and aggregation (de Faria e Silva et al., 2020a, 2020b), and does not need G factor correction, simplifying measurement. Data were blank subtracted before any data analysis using the corresponding buffer for each step (see Supporting Information). pEEM data were corrected for differences in instrument response using a correction factor calculated using a Spectral Fluorescence Standard Kit (Sigma, product No. 69336) (Resch‐Genger et al., 2005), for a restricted *λ*_em_ = 302–450 spectral range (this was because the standards only covered the 300–700 nm emission range). Following this, the Rayleigh scatter (RS) area was replaced with missing data, and the fluorescence signal smoothed using Savitzky–Golay filter. For most analyses, the smoothed spectra were normalized to the point of the maximum intensity. The extracted RS band (RS_||_) is the first order Rayleigh scattering spectrum, and from this, the area under the curve (RS volume) was calculated. Both of these parameters were used for qualitatively assessing protein aggregation. Descriptions of sample types, data collected, and datasets used for modeling are provided in Supporting Information: Table S2.

Chemometric analysis was performed using PLS_Toolbox8.2.1®, MATLAB (ver. 9.1.0), and in‐house written codes. Exploratory data analysis was carried out using ROBust principal component analysis (ROBPCA), which minimizes the effect of outliers (Hubert et al., 2005) compared to classical PCA. It was implemented using the Venetian blind method (four splits) for cross‐validation and the root mean square error (RMSE) values to select the optimum number of PCs. Quantitative modeling for DAR and percent of aggregates was implemented using unfolded PLS, u‐PLS, (Haaland & Thomas, 1988). Model performance was assessed by coefficient of determinations (*R* ^2^), RMSE, and relative error of prediction (REP). The elliptical joint confidence region (EJCR) test was used to compare the accuracy and precision of different models at a 95% confidence interval (Mandel & Linnig, 1957). iPLS was used for variable selection and it works by comparing u‐PLS performance with and without each variable (here each excitation wavelength), selecting variables that return lower cross‐validation errors (Nørgaard et al., 2000). The pooled relative standard deviation (RSD_P_) of SEC and DLS parameters was calculated using the RSD of values obtained from replicate measurements (see SI).

SEC was performed using a 300 × 7.8 mm mAb PAC‐SEC1 column (ThermoFisher) with a 5 µm particle size with an Agilent 1260 HPLC system equipped with a DAD detector. Solutions were filtered using a 0.20 μm Captiva PES filter and 10 µl of sample were injected in triplicate at 30°C with 50 mM Sodium Phosphate pH 6.8 + 300 mM NaCl buffer as the mobile phase, and a 0.8 ml/min flow rate. The important output parameter used for characterization and modeling was the %Agg. value which for this study was defined as: the ratio in % terms of the sum of the area under the curve (AUC) of the aggregate peaks (all those at Rt < 10 min) divided by the total peak area in the SEC chromatograms. Because no fragment peaks were observed and the buffer components peak (~14 min) was excluded, the percent of aggregates corresponded to 100‐(% Monomers). Here, the aggregate peaks occur between *Rt* = ~7 and 10 min. However, we do have to note that the %Agg. values here represent the soluble species and do not take into account large aggregates which might precipitate out or otherwise be lost. DLS data were collected at 20°C, after filtration (0.20 μm PES filter), using a Malvern Zetasizer Nano ZS (173°detection angle). Each sample was measured five times (each measurement was an average of 10 runs of 10 s duration) in disposable plastic cuvettes. Z‐average size (radius) and polydispersity index (PdI) were obtained by cumulants analysis while the hydrodynamic radius (*R*_h_) was extracted from the distribution fit (both from the Intensity PSD), using the Zetasizer software, ver. 7.13 (Malvern Panalytical). SDS‐PAGE was performed according to the BioRad TGX Precast Gels® specifications (see Supporting Information for details).

## 3 RESULTS AND DISCUSSION

Starting materials (IgG‐SM) and partially purified products (Pur‐ADC) were first characterized using conventional methods to measure aggregation by SEC, size by DLS, and DAR via UV‐visible absorbance spectroscopy. These data were then used to explain the observed spectral changes in the pEEM spectra before using multivariate data analysis to build predictive models for DAR and aggregation content.

### 3.1 Conventional reference measurements

#### 3.1.1 DAR quantification

**Figure 1:**
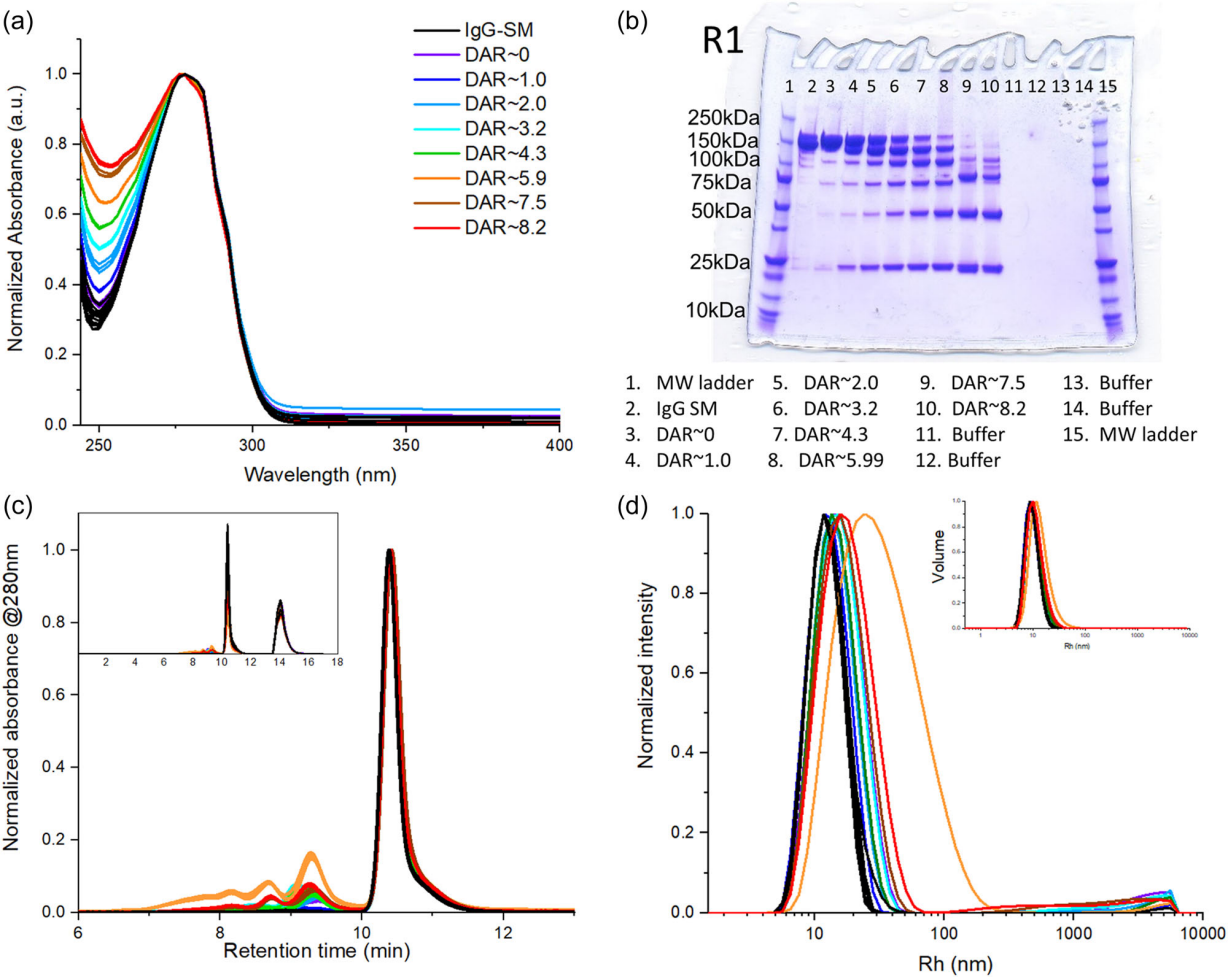
(a) Normalized absorbance spectra of purified products; (b) sodium dodecyl sulfate‐polyacrylamide gel electrophoresis (SDS‐PAGE) gels, coomassie blue stained, of Pur‐ADC samples (first reaction replicate only, see Supporting Information: Figure S4 for replicate measurements); (c) normalized size exclusion chromatography (SEC) chromatograms; (d) normalized DLS intensity obtained from analysis of starting materials (IgG‐SM) and purified products (Pur‐ADC); the data shown is the mean of the triplicate reactions carried out for each condition.

As all reactions were undertaken in cuvettes, we expected high recovery, however, absorbance spectroscopy suggested a decrease in total protein concentration over the course of the reaction (Figure 1a). Average absorbances of 1.08 ± 0.02 (IgG‐SM), 1.04 ± 0.01 (Red3‐IgG), and 0.98 ± 0.02 g/L (Alk4‐ADC) were measured, with a decrease due mostly to dilution via reagent addition. Recovery after purification was ~75% which was a significant protein loss experienced during handling and reconstitution of purified material which is to be expected with the small reaction volumes used. The final purified solutions used for measurements had a concentration (measured by absorbance) of 1.05 ± 0.02 g/L. DAR can be considered one of the most important CQAs because it determines the final product potency and stability. Absorbance spectroscopy is often used to determine DAR if drug and mAb have different absorption maxima (Chen, 2013; Wakankar et al., 2011). However, free drug is a problem if its absorbance spectrum overlaps that of the conjugated drug, which could lead to DAR overestimation. Here, we collected absorbance spectra from the purification washings to ensure complete free “drug” removal. DAR was calculated from the ratio of the concentrations of “drug” and mAb (see Supporting Information) which were determined using the extinction coefficients at the two wavelengths of maximum absorbance (Hamblett et al., 2004). It confirmed that varying degrees of conjugation, 1.0 ± 0.0 to 8.2 ± 0.1 DAR (Table 1), were achieved. The maximum number of conjugation sites should be eight, however, the slightly higher DAR value measured could be a result of small variations in protein absorption at 250–270 nm or possibly some conjugation to intrachain disulfide bonds (which would result in more than eight free thiols and higher DAR).

**Table 1:** Average sample data: nominal and PLS predicted DAR, percent of aggregates (from SEC), hydrodynamic radius, *R*_h_ (from DLS), and Rayleigh scatter (RS) volumes, see also Supporting Information: Figure S5

(TCEP) (M excess)	DAR		% Aggregates (from SEC)		*R*_h_ (from DLS)		RS volume (from EEM_||_)	
	Nominal	Predicted (EEM_||_)	IgG‐SM	Pur‐ADC	IgG‐SM	Pur‐ADC	IgG‐SM	Pur‐ADC
0 Control	0.1 ± 0.1	0.2 ± 0.4	0.8 ± 0.1	8.8 ± 4.0	6.3 ± 0.2	8.2 ± 0.1	19,250 ± 206	28,345 ± 912
1.25	1.0 ± 0.0	0.9 ± 0.3	0.8 ± 0.1	1.8 ± 0.5	6.4 ± 0.4	6.7 ± 0.1	20,220 ± 198	21,314 ± 382
2.5	2.0 ± 0.0	2.1 ± 0.1	0.8 ± 0.1	7.6 ± 2.3	6.4 ± 0.2	7.5 ± 0.4	19,822 ± 48	26,481 ± 548
5.0	3.2 ± 0.1	3.4 ± 0.1	0.8 ± 0.1	10.0 ± 2.6	6.4 ± 0.1	8.1 ± 0.4	19,877 ± 93	28,346 ± 498
7.5	4.3 ± 0.1	4.4 ± 0.0	0.7 ± 0.1	7.9 ± 2.2	6.3 ± 0.1	7.6 ± 0.3	19,629 ± 18	26,429 ± 487
10	5.9 ± 0.0	6.0 ± 0.1	0.7 ± 0.1	27.5 ± 1.1^a^	6.4 ± 0.1	19.4 ± 1.6^a^	25,546 ± 2559	65,798 ± 1687
25	7.5 ± 0.1	7.6 ± 0.2	0.8 ± 0.1	11.2 ± 0.9	6.7 ± 0.4	8.6 ± 0.1	22,647 ± 2448	29,523 ± 862
50	8.2 ± 0.1	7.9 ± 0.2	0.8 ± 0.1	13.3 ± 1.0	6.2 ± 0.1	9.5 ± 0.3	23,888 ± 1173	33,687 ± 844

*Note*: DAR was calculated from Pur‐ADC absorbance spectra, according to the procedure given in the Supporting Information and PLS predicted DAR was obtained from u‐PLS models of EEM_||_ after iPLS variable selection (calibration and validation data were included). More DLS data (e.g., Z‐avg.) is available in Supporting Information: Table S3. *R*_h_ was defined as the *R*_h_ calculated for the main peak (results are the average of three reactions measured 5 × 10 times). Abbreviations: ADCs, antibody‐drug conjugates; DAR, drug‐to‐antibody ratio; SEC, size exclusion chromatography.

^a^ In addition to the high DAR, these samples had a long hold time during purification, which might explain the higher aggregation.

#### 3.1.2 Physical characterization

SDS‐PAGE gels indicated that all reactions were relatively clean, while also giving information about attachment sites within the antibody (Figure 1b). The rationale behind this is the dissociation of the antibody into light and heavy chains (L and H) which are no longer covalently attached via disulfide bonds (because of conjugation). With the increased TCEP (and thus higher DAR), the amount of unconjugated IgG (HHLL, 150 kDa) decreased, with a concomitant increase in dissociated L (25 kDa), H (50 kDa), HL (75 kDa), HH (100 kDa), and HHL (125 kDa) species depending on the degree of conjugation and site of attachment. This agreed with the claim that inter H‐L di‐sulfide bonds were the first reduced under mild reducing conditions (Guo et al., 2014). The gels also showed good reproducibility between replicate experiments as demonstrated by the band patterns (Supporting Information: Figure S4). SEC‐HPLC (Figure 1c) aggregation data and DLS (Figure 1d) *R*_h_ data (Table 1 and Supporting Information: Table S3) showed that all IgG‐SM had very similar aggregation profiles (99.2 ± 0.1% monomer) and sizes. These measurements also showed that aggregation increased after purification (87.9 ± 13.3% monomer).

### 3.2 pEEM measurements

#### 3.2.1 Fluorescence spectroscopy

**Figure 2:**
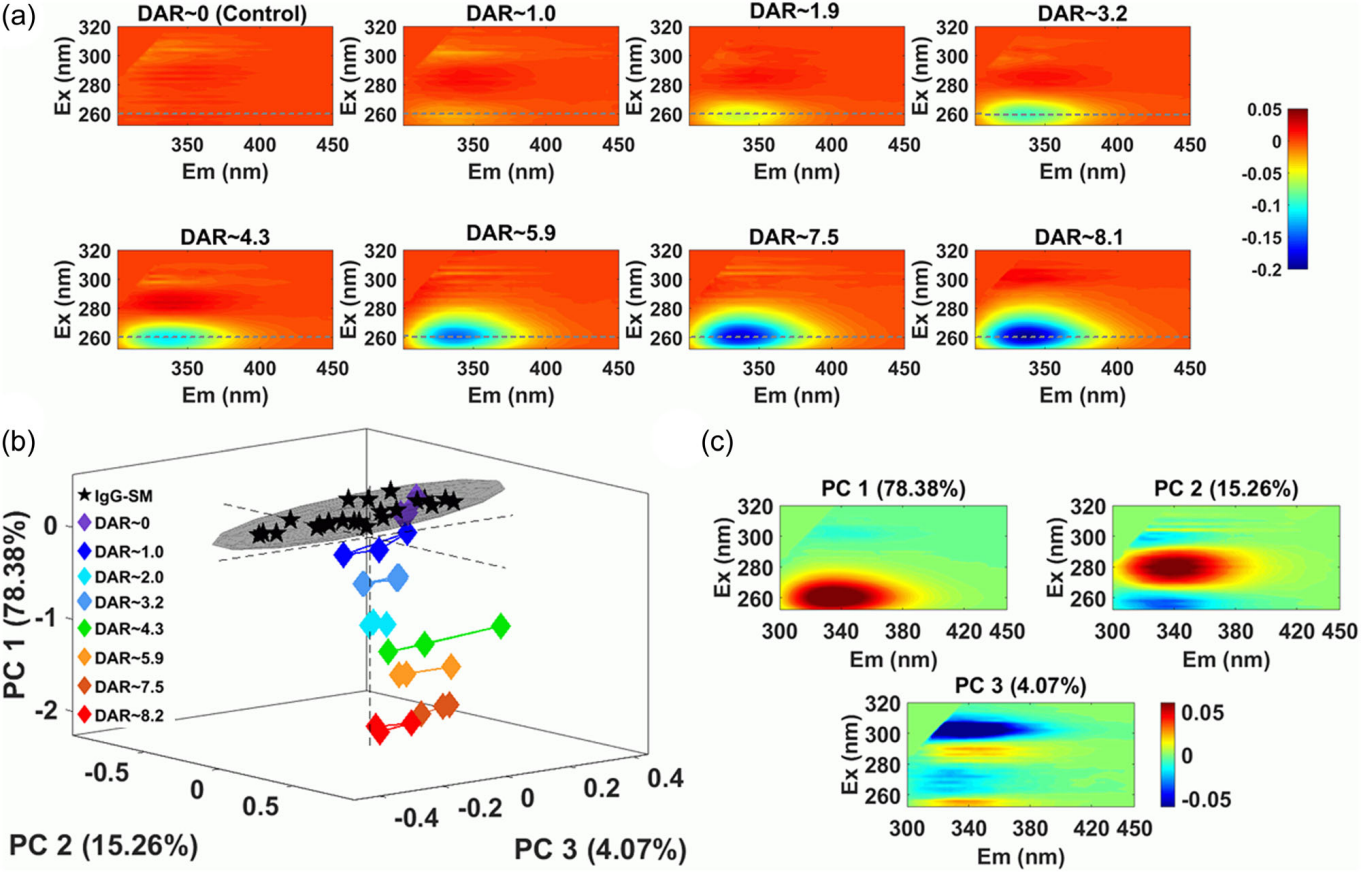
(a) Difference spectra (Pur‐ADC – IgG SM) calculated using normalized EEM_||_ spectra plotted over the 300–450 nm emission range (the dashed lines show the 260 nm excitation). The spectra used for calculation were the average from the triplicate reactions carried out for each reaction condition. ROBust Principal Component Analysis (ROBPCA), using Normalized EEM_||_ data from the Pur‐ADC and IgG SM sample set (*n*= 48); (b) scores and (c) loadings plots.

Normalized EEM_ǁ_ difference spectra showed significant intensity changes (at *λ*_ex_ < 260 nm) of up to 20% for the highest DAR samples (Figure 2a) which was probably related to secondary changes in fluorescence induced by varying Inner filter Effect (IFE) from light absorption of the attached linker, rather than large changes in intrinsic protein emission. IFE is usually considered a problem because it causes a nonlinear dependence between intensity and concentration. However, it is a valuable source of information about protein‐based samples because of the high sensitivity to changes in sample composition (Panigrahi & Mishra, 2019; Ryder et al., 2017) and can be incorporated into variance assays once identified and taken into account.

To better interpret the spectral changes and their significance we used ROBPCA, which generates loadings plots to provide information about the types of spectroscopic changes occurring and scores plots that provide data on the magnitude/significance of these changes. ROBPCA of IgG‐SM + Pur‐ADC samples (Table 2, Figure 2b,c) easily discriminated conjugated from nonconjugated mAb with most separation along PC1 (78% explained variance) which is caused by spectral differences at *λ*_ex/em_~260/340 nm due to increased absorbance by attached linker molecules (*λ*_max_ = 250 nm) and a corresponding decrease in Trp emission as DAR increases due to IFE induced by linker attachment. PC2 (15.26%) represents changes centered at *λ*_ex/em_~280/340 nm and seems to be related to changes in the directly excited intrinsic fluorophores (Trp and Tyr) but mostly Trp. Because PC2 scores decreased with DAR, this suggested some form of quenching via non‐radiative transitions. PC3 (4.07%) seems to represent the intrinsic protein variance related to small variations in concentration (~2%) as sample distribution was linear along PC3 and the scores showed a negative signal at *λ*_ex_ ~300 nm with very little change at shorter excitation wavelengths. The fact that IgG‐SM samples were distributed along PC3 also supports this view.

#### 3.2.2 DAR quantification

**Figure 3:**
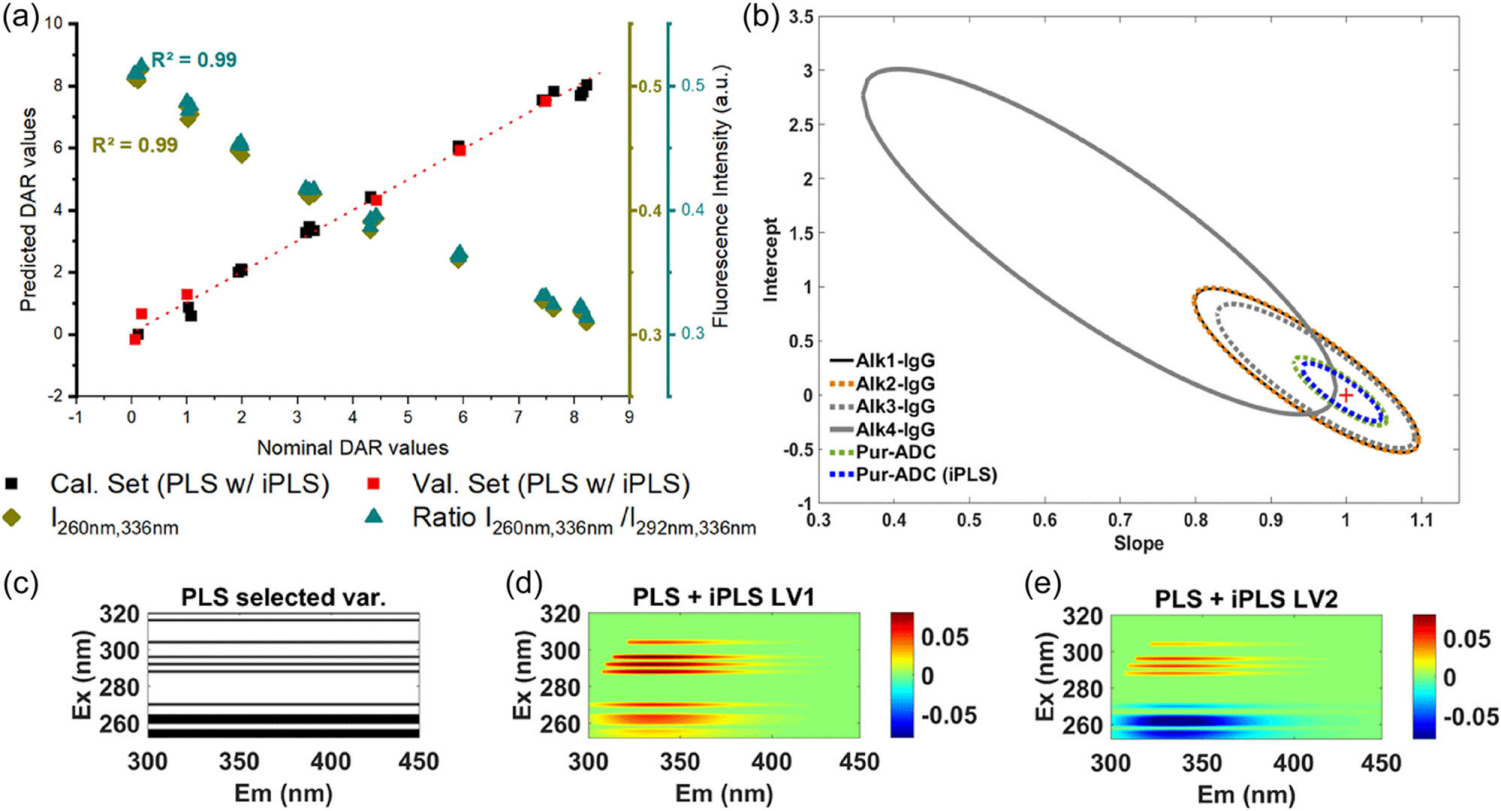
(a) Plots of nominal versus predicted drug‐to‐antibody ratio (DAR) values from u‐PLS modeling of all **Pur‐ADC** samples after iPLS variable selection. Linear regression results of nominal DAR values versus intensity at *λ*_ex/em_ 260/336 nm (green) and ratio between *λ*_ex_ 260/336 nm and 292/336 nm (blue). (b) Elliptical joint confidence region (EJCR) plot at 95% confidence level for the regression slope and intercept (1, 0) of predicted versus nominal DAR values of Pur‐ADC. (c) Selected wavelengths (in black) after iPLS variable selection for models built with **Pur‐ADC**. (d, e) LV1 and 2 loadings from u‐PLS models built using Pur‐ADC excitation emission matrix data after variable selection.

Based on these observations, we built a u‐PLS model (Table 3) for DAR quantification using the Pur‐ADC normalized pEEM spectra (Figure 3a) with samples split into calibration and validation sets using the Kennard‐Stone Algorithm. The best model obtained had relatively small error (RMSE < 6%), and then by using iPLS variable selection, it was possible to reduce the number of excitation wavelengths, facilitating shorter acquisition times while maintaining similar prediction performance (REP = 8%) as shown in the EJCR plot (Figure 3b). Both u‐PLS selected variables and ROBPCA loadings (Figure 3c–e) indicated that two main spectral regions contributed to the quantification model, thus one can build a simpler, more transparent DAR correlation model using single data points, for example, intensity at *λ*_ex/em_ = 260/336 nm, or better use a ratio measurement between *λ*_ex/em_ 260/336 and 292/336 nm. Both gave good correlations (*R* ^2^ = 0.99) with the nominal DAR. This demonstrates a key use of EEM measurements and chemometric analysis for quickly screening the full emission space to find simpler measurement options.

**Table 2:** Summary of ROBPCA EEM_||_ modeling results for: (1) All reaction/product samples, (2) only IgG‐SM samples, (3) only reduction intermediate samples (Red‐IgG), (4) combined model of IgG‐SM and Red‐IgG samples, (5) only alkylation intermediates (Alk‐IgG), and (6) IgG‐SM and Pur‐ADC samples.

ROBPC	All samples (*n* = 216)	IgG‐SM + Red‐IgG (*n* = 96)	Alk‐IgG^a^ (*n* = 96)	IgG‐SM (*n* = 24)	Red‐IgG (*n* = 72)	IgG‐SM and Pur‐ADC (*n* = 48)
1	98.55	91.65	71.42	73.29	91.12	78.38
2	1.20	2.87	21.50	18.93	2.68	15.26
3	0.06	1.46	2.99	1.07	1.71	4.07
4	‐	‐	0.49	‐	‐	0.44
Total variance	**99.81**	**95.98**	**96.41**	**93.29**	**95.51**	**98.15**

*Note*: The best model are shown in bold.

Abbreviations: ADCs, antibody‐drug conjugates; EEM, excitation emission matrix; ROBPCA, ROBust principal component analysis.

^a^ This model did not include control samples, which were considered outliers in the model because of the absence of linker.

**Table 3:** Summary of u‐PLS DAR prediction results using normalized spectra of alkylation intermediates and Pur‐ADC with and without iPLS variable selection

	Absorbance spectra				pEEM						
	Alk1‐IgG	Alk2‐IgG	Alk3‐IgG	Alk4‐IgG	Alk1‐IgG	Alk2‐IgG	Alk3‐IgG	Alk4‐IgG	Pur‐ADC		
Var. Sel.	**‐**	**‐**	**‐**		**‐**	**‐**	**‐**	**‐**	**‐**	**iPLS**	
RMSE Cal	0.49	0.48	0.53	0.91	0.61	0.61	0.53	1.53	**0.25**	0.21	
RMSE CV	0.71	0.66	0.75	1.42	1.30	0.98	0.76	3.18	**0.29**	0.23	
RMSE Pred (REP)	0.89 (28%)	0.91 (29%)	0.88 (28%)	1.72 (54%)	0.56 (18%)	0.68 (22%)	0.34 (11%)	1.15 (39%)	**0.19** (6%)	0.25 (8%)	
*R* ^2^ Cal	0.97	0.97	0.96	0.88	0.95	0.95	0.96	0.67	**0.99**	0.99	
*R* ^2^ CV	0.93	0.94	0.92	0.73	0.82	0.88	0.92	0.11	**0.99**	0.99	
*R* ^2^ Pred	0.94	0.94	0.94	0.90	0.96	0.97	0.99	0.90	**1.00**	0.99	

*Note*: In all cases, the total sample number was 24, which were split into calibration (*n* = 18) and external validation (*n* = 6) sets (the same samples in all cases). The best model are shown in bold.

Abbreviations: ADCs, antibody‐drug conjugates; DAR, drug‐to‐antibody ratio; pEEM, polarized excitation emission matrix; RMSE, root mean square error.

### 3.3 Aggregation analysis and prediction

**Figure 4:**
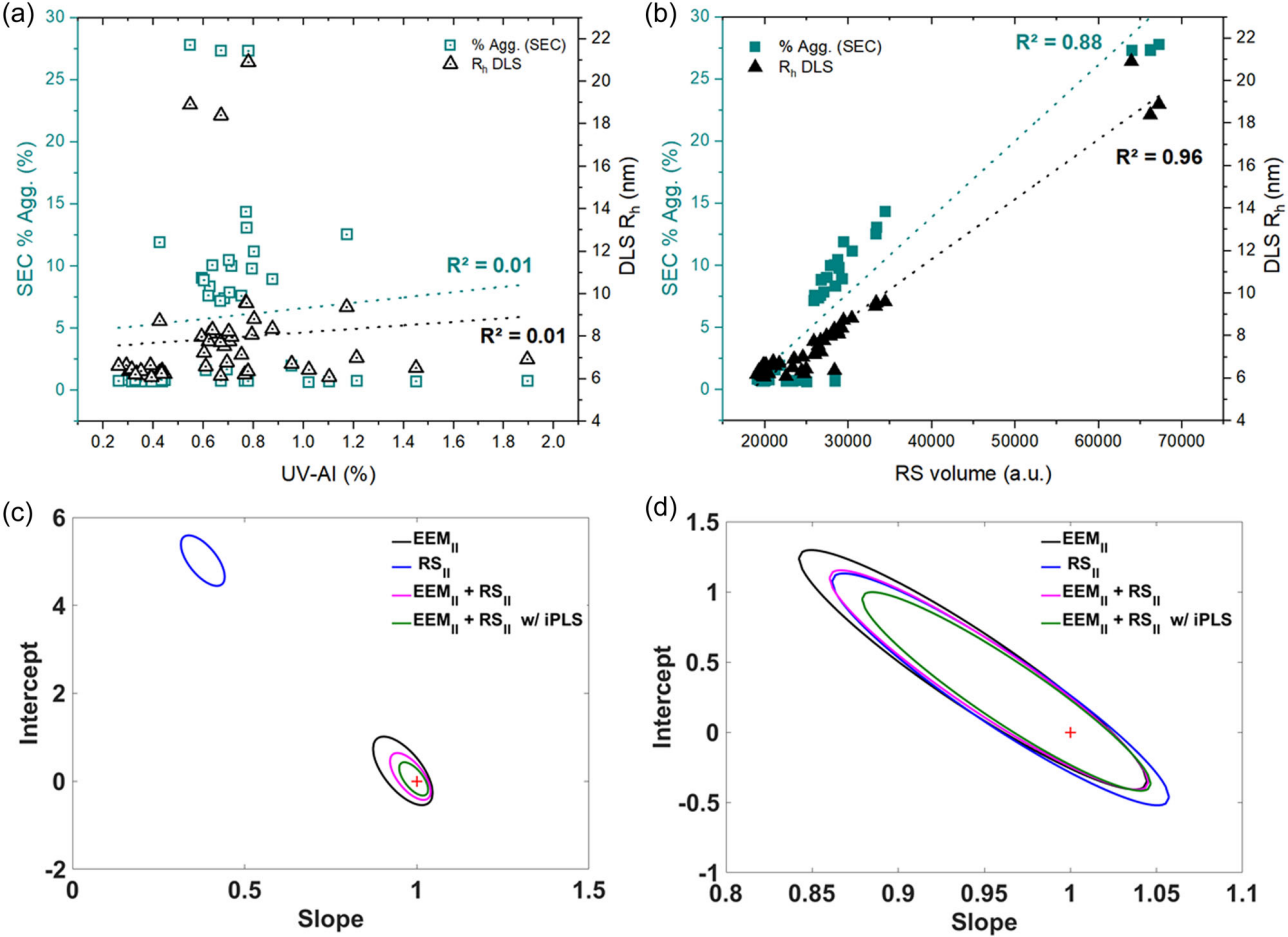
Scatter plots showing sample distribution, with % aggregation (as determined by size exclusion chromatography [SEC]) and *R*_h_ and (DLS) measurements plotted against: (a) UV‐AI, and (b) RS_||_ volume measurements. Linear fits are included for reference. Elliptical joint confidence region (EJCR) plots for u‐PLS regression models (using SM and Pur‐ADC sample sets and RS_||_ and EEM_||_ data) for prediction of: (c) %Aggregation (%Agg,), and (d) *R*_h_ values (main [small species] peak from distribution fit of the DLS data).

Aggregation is a critical CQA to be monitored during ADC production and it is thus important to monitor changes in both tertiary and quaternary structure. An increase in insoluble aggregates was indicated by a general rise in the ultraviolet aggregation index ($\mathrm{UV}-\mathrm{AI}={(A}_{350}/{(A}_{280}-A_{350}))\times 100$), 0.61 ± 0.43 → 1.35 ± 0.58 → 1.96 ± 0.76% for IgG‐SM, Red‐IgG, and Alk‐IgG (average values for all samples, *n*= 24), respectively (Wang & Roberts, 2010). After purification, UV‐AI decreased to 0.72 ± 0.15% which could be a result of either de‐aggregation or more probably selective aggregate loss during purification, since recovery was only ~75% (e.g., sedimentation of large aggregates). All the individual reactions apart from the control show similar trends and the complete data is available in the Supporting Information (Supporting Information: Table S5/Figure S9). Previously we showed (de Faria e Silva et al., 2020b) that UV‐AI had a poor relationship with SEC measured aggregation, which was probably due to its poor sensitivity for small soluble aggregates. Turbidity measurements (usually OD_350nm_) have been used to monitor protein aggregation with stress conditions(Ross & Wolfe, 2016). Here, there was no correlation between UV‐AI with either SEC or DLS *R*_h_ values (Figure 4a), which shows that UV‐AI is unsuitable for this type of sample/process where relatively low levels of soluble aggregates are present. However, there was a good correlation (*R* ^2^ > 0.9, Table 1, plot not shown) between *R*_h_ and % aggregates for the Pur‐ADC, which indicated that the issue for UV‐AI was sensitivity. RS_ǁ_ volume on the other hand, generated significantly better correlations to both *R*_h_ (DLS) and %Agg (SEC), *R* ^2^ = 0.96 and 0.88 respectively (Figure 4b), for the combined IgG‐SM and Pur‐ADC sample set. Considerably lower correlations (*R* ^2^= 0.44) were obtained for Z‐average which was unsurprising since the Z‐average size metric is unreliable for polydisperse samples with PdI > 0.1, (Bhattacharjee, 2016). Here, all the Pur‐ADC samples had PdI > 0.2. It is probably because of the ~30% (on average) *R*_h_ increase between IgG‐SM to Pur‐ADC (Supporting Information: Table S3) that we get good correlations with RS_ǁ_ volume measurements. In cases where the distribution fits showed a second larger species, it was present only in relatively small quantities (<3% in the Pur‐ADC) and as such seem to have a low influence on the model. However, these reference DLS size measurements need to be investigated further in future studies.

u‐PLS predictive models (Table 4) for quantification/prediction of %Agg (SEC) and *R*_h_ (DLS) using EEM_ǁ_ and RS_ǁ_, showed that *R*_h_ prediction was better. This suggested that the presence of species which were not detected by SEC (e.g., noncovalent aggregates or very large particles), had a significant impact on EEM_ǁ_ and RS_ǁ_ spectra. Noncovalent aggregates could be either agglomerates or reversible aggregates, however, we have no data available to discriminate between the two. The higher errors obtained for %Agg prediction (REP > 18%) can also be related to greater SEC measurement errors (RSD_P_ = 4.2%) compared to DLS (2.3%). Other error factors were possible sample changes caused by an extra freeze‐thaw cycle, additional sample handling, and the time delays making SEC measurements.

**Table 4:** Summary of u‐PLS modeling results obtained for percent of aggregate and *R*_h_ quantification using the fluorescence signal from EEM_ǁ_, the RS_ǁ_ (not normalized), and the combined fluorescence and scatter signal.

IgG SM + Pur‐ADC (*n* = 48, cal. = 36/val. = 12)^a^									IgG SM + Red3‐IgG + Alk4‐IgG + Pur‐ADC (*n* = 96, cal. = 72/val. = 24)^a^			
	% Agg. (from SEC)				*R*_h_ (from DLS)				*R*_h_			
	**EEM**_**||**_	**RS**_**||**_	**EEM**_**||**_ + **RS**		**EEM**_**||**_	**RS**_**||**_	**EEM**_**||**_ + **RS**_**||**_		**EEM**_**||**_	**RS**_**||**_	**EEM**_**||**_ + **RS**	
			**‐**	**iPLS**			**‐**	**iPLS**			**‐**	**iPLS**
RMSE Cal	1.39	4.63	0.94	0.67	0.74	0.70	0.67	0.57	1.42	0.70	0.51	0.46
RMSE CV	2.28	4.76	1.18	0.93	1.32	0.72	0.78	0.71	1.78	0.72	0.70	0.61
RMSE, Pred	1.91 (31%)	4.86 (79%)	1.28 (21%)	1.13 (18%)	0.63 (9%)	0.59 (9%)	0.54 (7%)	0.47 (6%)	1.61 (21%)	0.81 (11%)	0.60 (8%)	0.49 (6%)
*R* ^2^ Cal	0.96	0.87	0.98	0.99	0.94	0.95	0.95	0.97	0.62	0.91	0.95	0.96
*R* ^2^ CV	0.89	0.88	0.97	0.98	0.82	0.95	0.94	0.95	0.42	0.90	0.91	0.93
*R* ^2^ Pred	0.94	0.90	0.99	0.99	0.97	0.98	0.99	0.99	0.67	0.91	0.97	0.97

*Note*: For u‐PLS modeling EEM spectra were normalized to maximum intensity, EEM with RS were normalized to λex/em 294/336 nm, which is a data point with high intensity value and low StDev. Abbreviations: ADCs, antibody‐drug conjugates; EEM, excitation emission matrix; RMSE, root mean square error.

^a^ The same samples were used for calibration and prediction in each sample group to enable comparison between various measurements/parameters.

Aggregate content (%Agg. from SEC measurements) was better correlated to fluorescence than to scatter signals (but best when both signals were used), which was probably due to the weaker scatter contribution of the nm‐sized soluble aggregates (most samples were composed of monomers, dimers, and trimers according to SEC), and also because of the noisy/variable absolute scatter signal measured. This is clearly seen in EJCR plots (Figure 4c) which confirmed that the model using both EEM_ǁ_ + RS_ǁ_ data with variable selection was best. Here, with a relatively large sample set and using a mAb which better represents therapeutically relevant molecules, pEEM can be used for the quantification of soluble aggregates (as determined by SEC measurements) down to ~1% (Table 4) which is significantly better than demonstrated previously for a polyclonal IgG (de Faria e Silva et al., 2020b).

pEEM spectral variance correlated better with *R*_h_, with the RS_**||**_ based models being slightly better than those using EEM_ǁ_ spectra (Table 4). The major issue with using non‐normalized RS data for modeling aggregation was noise and poor reproducibility because the model relied on absolute intensity changes. We attempted to quantify aggregation using normalized RS_**||**_ (by either band maximum or area), but this produced worse results (data not shown). Despite this, the error was relatively low and could be improved by spectral averaging. Another solution is to simply use normalized full pEEM spectra to minimize unwanted signal fluctuations (fluorescence and RS), which should be a more reproducible source of size‐related changes. Here, a specified spectral data point (*λ*_ex/em_ 294/336 nm, point of maximum fluorescence intensity) was used for normalization. Using this data resulted in better *R*_h_ models with good correlation coefficients (*R* ^2^ > 0.95) and a 2%–3% decrease in REP for EEM_ǁ_ + RS_ǁ_ compared to RS_ǁ_. Larger improvements were obtained for aggregate content (Table 4) using EEM_ǁ_ + RS_ǁ_, with *R* ^2^ > 0.98 and a REP decrease of 10%–13%. iPLS selected variables and loadings (Supporting Information: Figure S6) showed that areas of the fluorescence signal and scatter band were important for size prediction of (Figure S6d,g,h) and aggregate content (Supporting Information: Figure S6o,l,p), with the RS signal always being the strongest contribution. EJCR plots (Figure 4d) confirmed that the best model used both EEM_ǁ_ + RS_ǁ_ data and variable selection.

## 4 REACTION MONITORING

### 4.1 Spectral changes

**Figure 5:**
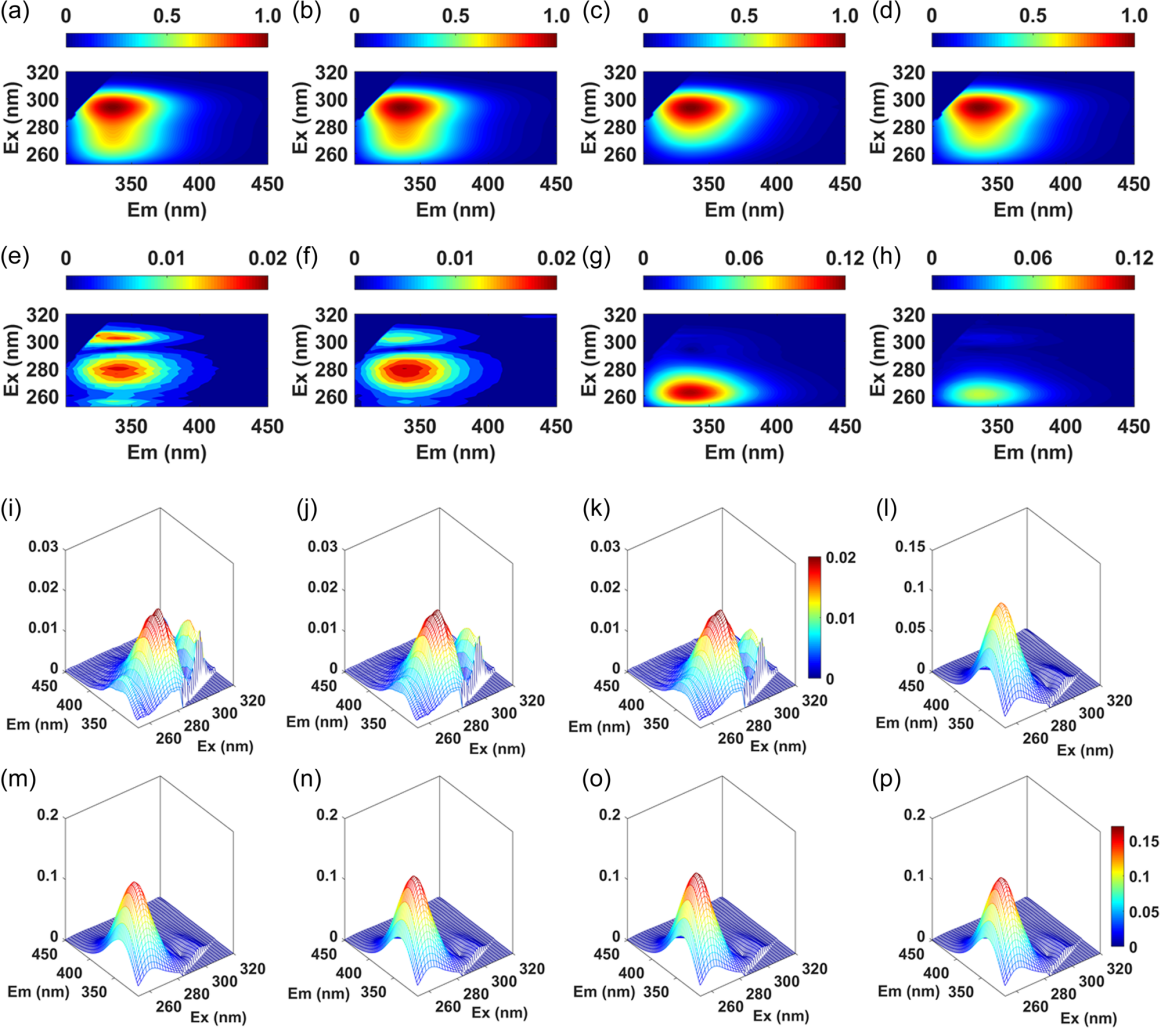
Rows 1 and 2: Mean (top) and Std.dev (bottom) spectra calculated for IgG SM (a, e), Red‐IgG (b, f), Alk‐IgG (c, g), and Pur‐ADC (d, h) showing the variance between the 24 reaction solutions in each of these four datapoints during the reaction. Rows 3 and 4: Cumulative standard deviations calculated from addition of extra process step samples to the starting material sample set (*n*= 24): (i)+Red1‐IgG (*n*= 48); (j)+Red2‐IgG (*n*= 72), (k)+Red3‐IgG (*n*= 96), (l)+Alk1‐IgG (*n*= 120), (m)+Alk2‐IgG (*n*= 144), (n)+Alk3‐IgG (*n*= 168), (o)+Alk4‐IgG (*n*= 192), and (p)+Pur‐ADC (*n*= 216). This shows the amount of signal variation available for modeling over the process, and how the scatter contribution decreases during the alkylation step. See Supporting Information: Table S2, for details of the sample sets.

To investigate reaction‐dependent spectral changes, we collected pEEM spectra every 45 min. during the reaction (nine measurements in total): one IgG starting material (IgG‐SM), three during reduction (Red1/2/3‐IgG), four during alkylation (Alk1/2/3/4‐IgG) and one of the partially purified product (Pur‐ADC). The mean (Figure 5a–h) and standard deviation spectra (Figure 5i–p) calculated from normalized spectra at each timepoint showed good reproducibility between IgG‐SM (Figure 5a,e), and no major spectral changes during reduction (Figure 5b,f). Changes were more significant after linker addition (Figure 5c,g) and this carried through to the Pur‐ADC (Figure 5d,h) although it was smaller presumably because the unreacted free drug had been removed. The relative standard deviation (RSD_EEM_), see Supporting Information for explanation, for all starting materials (*n* = 24) and all reduction intermediate samples (*n* = 72) was 1.8%, with a maximum of 2% for a single data point, and a significantly higher variance among alkylation intermediates and purified product (EEM_RSD_= 8.8 and 4.4%, *n* = 96 and 24, respectively). These were significant emission changes compared to 1% changes previously obtained for control measurements (de Faria e Silva et al., 2020b). It was not possible to collect more spectra during the reaction because the scanning‐based spectrometer took ~7 min. to collect one full spectrum, which limited the number of sample points available for analysis and analysis of reaction rates.

We assessed the changes at each reaction step using StDev calculated after successively adding data collected at each data point for example, for Step 3 of reduction StDev was calculated using IgG‐SM + Red1‐IgG + Red2‐IgG + Red3‐IgG samples (Figure 5). This again shows that alkylation was responsible for most of the reaction spectral variance, which increased from 8.2% to 11% (EEM_RSD_) from the first to last sampling point. The equivalent variance (i.e., reproducibility), between replicates of same reaction at a specific timepoint, was much lower (<2%). Overall, we can say that the spectral changes induced by alkylation were large, significant, and thus suitable for ROBPCA and quantitative modeling (Table 2).

**Figure 6:**
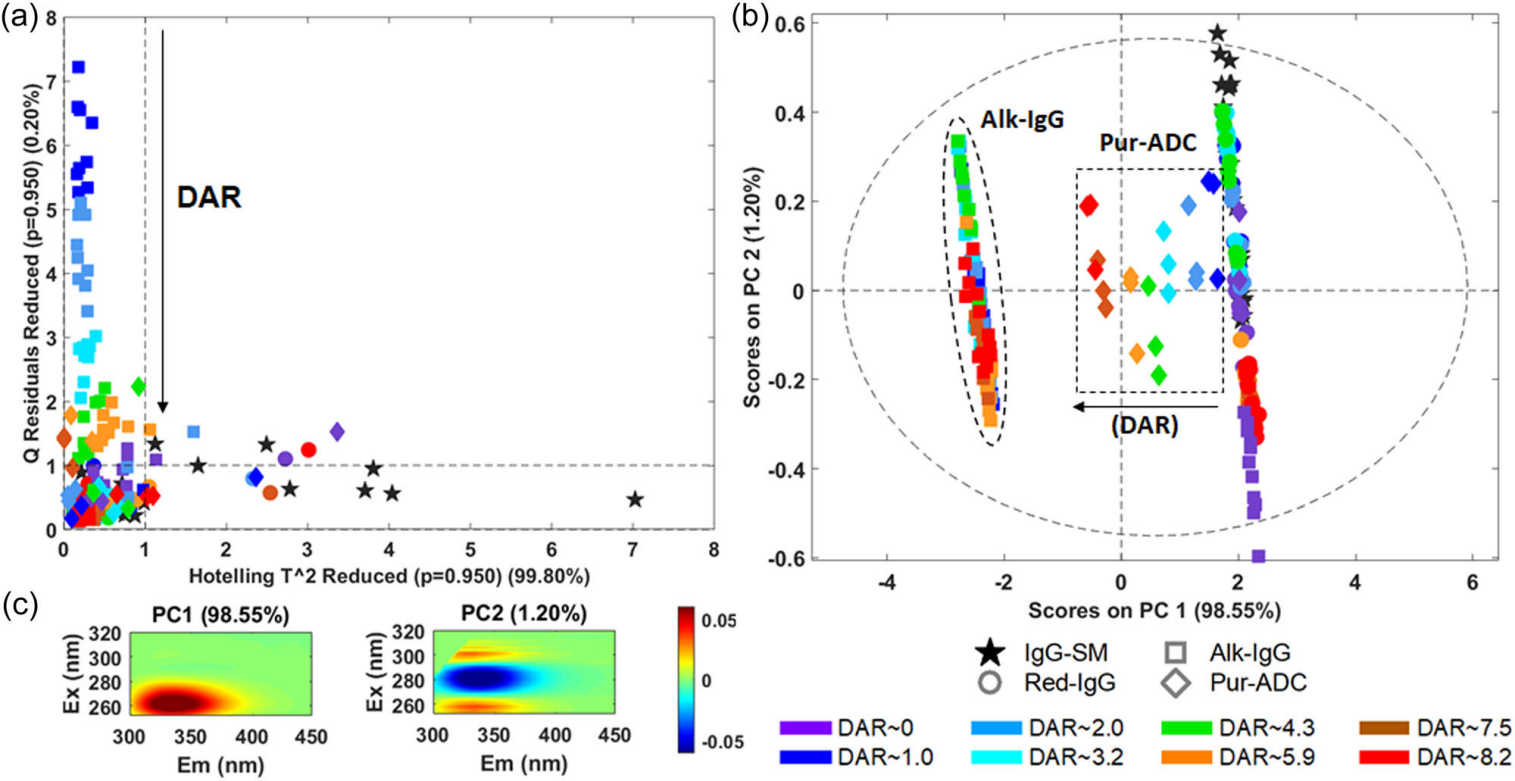
Results of ROBust principal component analysis (ROBPCA) analysis of EEM_||_ data set (IgG‐SM + Red1,2,3‐IgG+Alk1,2,3,4‐IgG, Pur‐ADC, *n*= 216). (a) Q residuals versus hoteling (outliers) plot, (b) PC1 versus PC2 scores; and (c) refolded loadings plots. The symbols represent the reaction stage (pentagram, circle, square, and diamond for IgG‐SM, Red‐IgG, Alk‐IgG, and Pur‐ADC, respectively) and colors the drug‐to‐antibody ratio (DAR) of final Pur‐ADC as indicated by the legend. The boundaries for Alk‐IgG and Pur‐ADC were included as a visual guide to show the two groups.

ROBPCA (Figure 6) was then used to better understand the source of these spectral changes and three ROBPCs were required to explain the spectral variance when models were built using all samples. This ROBPCA model contains more complex samples than that depicted in Figure 2 and the presence of excess unbound linker had the largest impact. The outliers plot (Figure 6a) showed the significantly different samples and indicated some IgG‐SM samas outliers (bottom‐right and top‐left quadrants), but also some of the alkylation intermediates for reactions producing a lower DAR. The latter is probably associated with changes in absorption/emission of free and linked drug, as there seem to be a decrease in Q Residuals with increasing DAR. The separation along PC1 (Figure 6b) is largely associated with changes in emission induced by the presence of linker (Figure 6c), which explained the separation of IgG‐SM and Red‐IgG groups from Alk‐IgG (conjugated + free linker) and from Pur‐ADC (varying DAR). ROBPC2, which only explained 1.20% of variance, represents a decrease in emission intensity, and thus probably represents small concentration and aggregate related variation arising from sample handling and other factors rather than significant structural change. This was because the tertiary structure of the starting material, reduced mAb, and the DAR 0 product should be similar. Thus, a global ROBPCA model containing all samples does not clearly show the reaction induced spectral changes very clearly.

The scores obtained for the two models (IgG‐SM + Red‐IgG and Alk‐IgG) were plotted (Supporting Information: Figures S7 and S8) against reaction timepoint showing the trajectory followed by the different reaction conditions which lead to different DAR products. ROBPC1 and 2 of IgG‐SM + Red‐IgG indicated, as expected, very small changes in IgG emission during reduction, and highlighted the starting materials as the main sources of variation. Because the variance among IgG and Red‐IgG was rather small (EEM_RSD_ < 2%), it is possible that part of the changes modeled are related to the instrument and/or measurement errors. For the alkylation process (Alk‐IgG samples), PC2 showed the clearest correlation with DAR. It suggested (Supporting Information: Figure S7F) a possible combination of IFE at 310 nm and changes in Trp local environment (from less to more hydrophobic), with increasing DAR, which might be associated with the amount of conjugated/free linker in solution which agreed with observations (Figure 2).

We attempted DAR prediction using pEEM spectra collected during Alkylation, before purification (Table 2). Here, absorbance spectroscopy was ineffective because of spectral overlap between free and conjugated linker, which resulted in very small spectral differences between in‐reaction samples with different DAR (the same amount of linker was added to all reactions). u‐PLS results suggested however, that there were small variances in pEEM spectra of the alkylation intermediates (Alk1–4), which correlated with DAR. Alk3‐IgG had a better correlation with DAR (*R* ^2^ > 0.92, REP = 11%) compared to Alk1/Alk2 because it was later in the reaction whereas the poorer correlation obtained with Alk4‐IgG seems to be caused by interference from addition of the reaction quencher, NAC. When Alk1‐4 absorbance spectra were used for DAR quantification, fairly good calibration results were obtained (relative error of calibration [REC] = 11%), but prediction errors were significantly worse (REP > 28%) compared to pEEM. Quantification here seems to be based on small absorbance changes at ~310 nm (and also IFE) due to loss of conjugation in the maleimide linker (–C=C–C=O → –CH–CR–C=O) after alkylation (Liu et al., 2013), with the high quantification errors caused by increased scattered light at ~310 nm.

Overall, these results suggested that EEM_ǁ_ was the better reaction monitoring option because of significant alkylation induced spectral changes. However, when we looked at the normalized score changes (Supporting Information: Figure S8) there was very little change from Alk1 to Alk3 which suggests that alkylation was faster than anticipated and was nearly complete before the first pEEM measurement was completed.

### 4.2 Physical stability (in reaction)

Previously we assessed polyclonal IgG solution quality using pEEM which indicated that most of the variance originated from longer wavelength emission (de Faria e Silva et al., 2020b). Here, long wavelength emission did not play a role in discriminating solutions according to aggregation which suggested that the previous observations were related to lower purity and higher variability of polyclonal IgG. The emission ratio between solvent exposed and buried Trp residues (I_350_/I_330nm_ using 296 nm excitation) is commonly used to assess protein stability (Beckley et al., 2013). Here, the ratio did not correlate with *R*_h_, which is clear evidence that there were no major structural changes like unfolding. As expected, however, the reaction/purification did induce some physical sample changes as observed by DLS in the reported *R*_h_ (Table 1) and Z‐average size values (Supporting Information: Table S3).

Although disulfide bond reduction could increase flexibility, and linker addition causes a small increase in product mass (~3% for a DAR of 8) these did not cause large changes in the *R*_h_ values: *R*_h_(IgG‐SM) = 6.4 ± 0.3 nm, *R*_h_(Red3) = 6.1 ± 0.1 nm, and *R*_h_(Alk4) = 6.7 ± 0.1 nm (Supporting Information: Table S3). However, there were very significant changes in derived count rates and PdI (~0.1 → ~0.5 → ~0.7 → ~0.2) and for IgG‐SM, Red3‐IgG, Alk4‐IgG, and Pur‐ADC, respectively (Supporting Information: Table S3). This suggested the formation of loosely bound reversible aggregates during the intermediate stages. After purification (Pur‐ADC samples) *R*_h_ and variability (9.4 ± 4.0 nm), increased, but there was also a significant drop in PdI compared to the intermediates. This indicated that the reversible aggregates formed earlier had broken down, and that the protein product may be somewhat aggregated compared to the mAb starting material. This was probably due to reduced stability caused the attached hydrophobic small molecules and variable DAR, and/or via the stresses of purification and extra sample handling. Similar trends were observed with RS volume, but these had higher measurement error (Supporting Information: Figure S5). *R*_h_ and RS volume did not correlate (*R* ^2^ < 0.5) and, poor u‐PLS regression results were obtained when using RS bands for size prediction.

While u‐PLS results showed similar *R*_h_ prediction performance using IgG‐SM and Pur‐ADC samples (EEM_ǁ_, RS_ǁ_, and combined spectra), size prediction results were better when two other reaction samples (Red3‐IgG, Alk4‐IgG) were added to the model. This was probably due to a larger sample set size and greater protein size variability when these samples were included. Overall, use of full (EEM + RS)_ǁ_ spectra enabled size prediction with low errors (REP < 8%) and good correlation with nominal values (*R* ^2^_Pred_= 0.97), implying a more robust size change assessment for complex in‐reaction samples.

### 4.3 Reaction end point determination

One goal of reaction monitoring is to accurately determine reaction end points, and this usually involves collecting multiple spectra throughout the reaction and then extracting kinetic and end‐point data. Here, the long spectral collections (because of th scanning spectrometer design) times coupled with the fast reaction kinetics, prevented this and thus we investigated a different approach. We used nonlinear, support vector machine (SVM) classification (Supporting Information: Table S4) to quantitatively assess if these low numbers of EEM_ǁ_ measurements could classify samples according to reaction stage and the ultimate product DAR (i.e., predict endpoint for different performing reactions). Samples were split into calibration and validation sets and 10 different classes were used for classification: IgG‐SM and reaction intermediates/Pur‐ADCs from reactions producing low (1.0–2.0), medium (3.2–4.3), and high (5.9–8.2) DAR. The classification errors indicated a good performance for successfully classifying both reaction stages and DAR (errors lower than 10% for all the classes) for the medium and high DAR ranges. The low DAR‐related classes (and the IgG‐SM) showed lower specificity, which was probably due to the very small spectral changes induced by the lower number of linker molecule attachments.

## 5 CONCLUSIONS

Using non‐destructive pEEM measurements for reaction monitoring of the important mAb linker conjugation process and product variance analysis has been demonstrated. Here, although the model reaction was limited to looking just at the linker addition, in small scale, and with insufficient time resolution, it does show the very significant spectral differences between each stage in the reaction process. Although spectral changes were relatively small (EEM_RSD_= 10 for all samples, *n* = 216), they were significant and reproducible. By using the full pEEM spectral information, that is both the scatter and fluorescence signals, one is able to build quantitative models for predicting DAR using the alkylation intermediates (*R* ^2^ > 0.90 and REP 11%) and that correlate with aggregation and size (*R*_h_, *R* ^2^_PRED_= 0.97 and REP = 6%) parameters extracted from SEC and DLS measurements, respectively. We also showed that simple UV‐AI measurements were poorly correlated with both %Agg. from SEC, and DLS‐derived size parameters, confirming its unsuitability for ADC reaction monitoring with these levels of soluble aggregates.

For reaction monitoring, the spectral profile changes observed here although small, were still significant considering that we only used normalized data. The u‐PLS modeling showed that it was possible to generate in‐process accurate correlations for both size and aggregation parameters when using the combined scatter and fluorescence signals. For wider application using payload molecules with different absorption spectra that overlap less with the protein absorption spectra, and potentially more with protein emission spectra, we suggest that fluorescence spectral changes will be larger and thus easier modeled using these techniques. Larger signal changes should make DAR quantification and reaction monitoring easier to implement and potentially more accurate and this is the focus of future studies.

Although fluorescence and pEEM are not currently, widely used analytical techniques for monitoring ADC synthesis and manufacturing processes (it is mostly used to assess the effect of conjugation on higher order structure and stability [Turecek et al., 2016; Wakankar et al., 2011]), these results are very promising. However, this alkylation reaction was too fast for this type of spectrometer and was substantially complete by 10 min when the first in‐process pEEM data was collected. For more accurate, continuous in‐process monitoring we require either faster data acquisition using spectrometers with multichannel detectors or slower reactions, both of which are being investigated, along with the use of more therapeutically drug linker moieties. Overall, this use of the full pEEM measurement shows considerable promise as a robust PAT tool for ADC manufacturing.

## AUTHOR CONTRIBUTIONS

**Ana L. de Faria e Silva**: Conceptualization; designed and performed the experiments; analyzed the data; prepared all the figures; wrote; and edited the manuscript. **Alan Ryder**: Conceptualization; supervision; funding acquisition; wrote and edited the manuscript.

## CONFLICT OF INTEREST

The authors declare no conflict of interest.

## Supporting information

Supplementary information.

## Data Availability

The data that support the findings of this study are available from the corresponding author upon reasonable request.
