# Supplementary material for: Analyzing protein conjugation reactions for antibody‐drug conjugate synthesis using polarized excitation emission matrix spectroscopy
Source: Biotechnol Bioeng. 2022 Sep 28;119(12):3432–46. doi: 10.1002/bit.28229 (PMC9828061; doi:10.1002/bit.28229)
Supplement: Supplementary file 1 — Supplementary information. [file BIT-119-3432-s001.docx]

**Analyzing protein conjugation reactions for Antibody-Drug Conjugate synthesis using** **polarized Excitation Emission Matrix spectroscopy.**

Ana Luiza de Faria e Silva and Alan G. Ryder.*

Nanoscale BioPhotonics Laboratory, School of Chemistry, National University of Ireland, Galway, H91 CF50, Ireland.

# Supplemental Information

**Pooled relative standard deviation (RSD_P_):**

$RSD_{P}=\sqrt{\frac{{(\text{n}}_{i}-1).R{SD}_{i}}{{(\text{n}}_{i}-1)}}$ , where n is the number of replicate measurements for each sample.([ISO et al., 1995](#_ENREF_6))

**Relative standard deviation (RSD_EEM_):** Calculated as the area of StDev spectra divided by mean spectra of all IgG-SM as follows: $RSD{}_{EEM}=\left( AUC{EEM_{stdev}}/{AUC EEM_{mean}} \right)$*100, calculated using norm-EEM.

## Reduction reaction condition screening.

Since di-sulfide bond reduction is a key step in determining the degree of modification, and we required a diversity of product DAR, we carried out multiple test reactions to define the best reduction conditions to achieve different levels of conjugation. For this, the mAb was mixed with various TCEP concentrations (0, 2.5, 5.0, 7.5, 10, 25, 50 molar excess) at 20°C and 37°C and the Ellman’s test was then undertaken to determine the number of free thiols.([Ellman, 1959](#_ENREF_2)) Human IgG1 contains 12 intra- and 4 inter-chain bridges, and it is the later which are the most susceptible to reduction under native conditions because of greater solvent exposure.([Liu & May, 2012](#_ENREF_7)) Thus, one expects reduction of a single IgG molecule to produce up to eight free sulfhydryl’s which are available for conjugation (two for each disulphide bond).

Thiol quantification by Ellman’s method is based on the reaction of DTNB (5,5’-dithio-*bis*-(2-nitrobenzoic acid) with a thiol to form a thiol-reagent conjugate with a concomitant release of one 5-thio-2-nitrobenzoic acid (TNB) molecule per available thiol (Figure 1C). Quantification was done using correlation with a standard curve of known sulfhydryl concentration.([Ellman, 1959](#_ENREF_2); [Hermanson, 2008](#_ENREF_5)) The absorbance spectra and standard curve are shown in Figure S1A/B. The DTNB reagent (5,5'-dithiobis-(2-nitrobenzoic acid) and cysteine*HCl*H_2_O (used for calibration plot) were prepared in the same PBS/EDTA reaction buffer. But, because TCEP can reduce the Ellman’s reagent producing TNB producing erroneous readings,([Han & Han, 1994](#_ENREF_4)) the TCEP was removed from the reaction mixtures by filtration using Amicon filters (10kDa cut-off). For the test, Ellman’s solution was mixed with the test samples, the solutions incubated at room temperature for 15 min, and the concentration of free sulfhydryl was determined using measuring absorbance at 412 nm and using a calibration plot of known cysteine concentrations. (Figure S1).


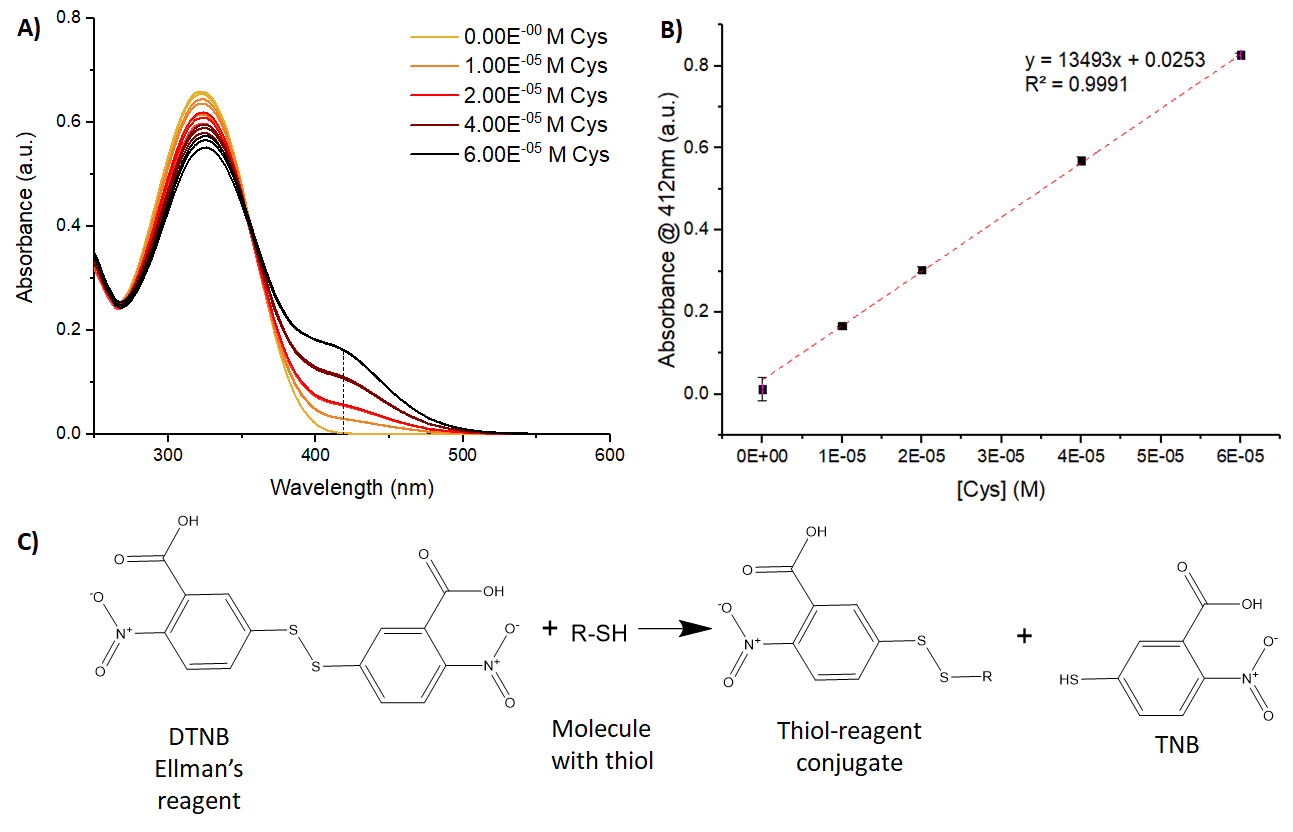


**Figure S1**-(A) Absorbance spectra of cysteine standard at various concentrations; (B) standard curve used for the quantification of free sulfhydryl by the Ellman’s method; and (C) schematic of chemical reaction between the Ellman’s reagent DTNB and the molecule with thiol.*(*[*Hermanson, 2008*](#_ENREF_5)*)*

Table S1 summarizes the results obtained from the reduction reaction screening. Two temperatures were tested, but we decided to start this study with the lower temperature because this should produce a slower reaction rate.

**Table S1:**  Results of free –SH quantification using the Ellman’s method carried out on the mAb containing solutions using different temperatures indicating the number of free thiols generated after reduction with various concentrations of TCEP. The values are the mean and StdDev of reactions carried out in triplicate.

| **Molar excess of TCEP** | **20 °C** | **37 °C** |
| --- | --- | --- |
| Control | 0.3±0.3 | 0.0±0.1 |
| 2.5 | 1.8±0.2 | 3.0±0.1 |
| 5.0 | 1.3±0.2 | 2.0±0.0 |
| 7.5 | 2.6±2.2 | 3.8±0.3 |
| 10 | 3.9±0.1 | 5.2±0.1 |
| 25 | 8.5±0.1 | 8.0±0.3 |
| 50 | 10.7±0.3 | 10.3±0.7 |


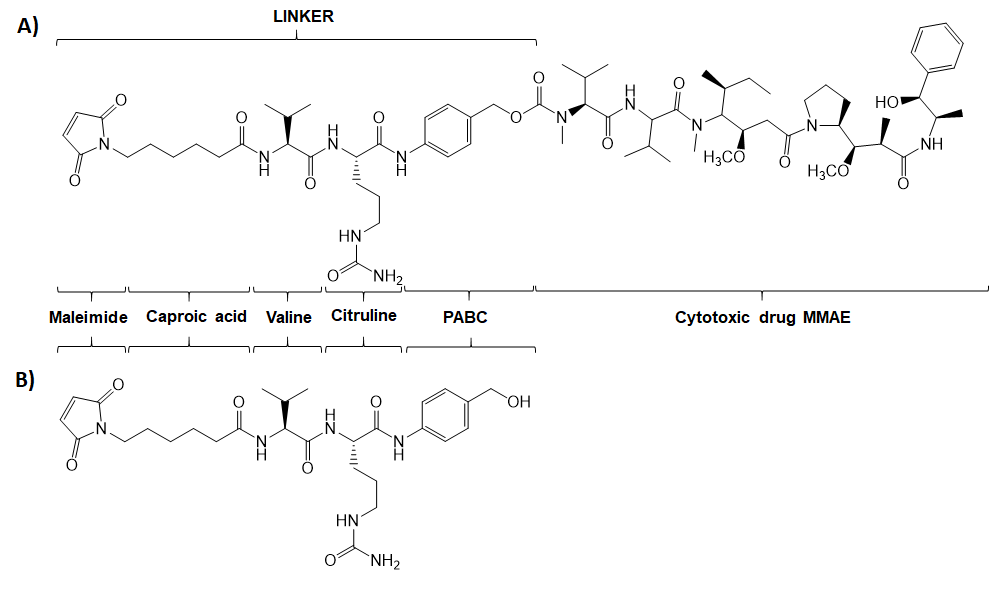


**Figure S2*:*** Chemical structure of (A) vc-MMAE: a common drug-linker used in some marketed ADCs; and (B) the non-toxic “surrogate” molecule used in this study. The difference when compared to the vc-MMAE drug-linker is the absence of the cytotoxic drug (here MMAE).





**Figure S3:** (A) IgG conjugation reaction scheme showing IgG structure, position of interchain disulphide bonds, which are the possible sites of conjugation, and the analytical measurements undertaken over the reaction. Reaction scheme of: (B) the reduction of disulphide bonds by TCEP, and, (C) addition of the drug to the free thiol. ([Hermanson, 2008](#_ENREF_5))

**mAb preparation & buffer exchange:** The mAb was shipped frozen in a large 1L batch (~45 g/L) which was stored at –70°C prior to aliquoting. This large batch was then carefully defrosted and aliquoted into smaller vials (2, 15, 50 mL) and containers suitable for single experiments and then all vials (>500) were then refrozen and stored at –70°C until required for individual experiments.

Phosphate buffer pH=7.0 was prepared from NaH_2_PO_4_ and Na_2_HPO_4*_7H_2_O using standard formulas with 150 mM NaCl, 10 mM disodium EDTA dehydrate, added as required. For buffer exchange, a 15mL Amicon ultra centrifugal filter was conditioned with 10 mL of buffer by centrifugation for 10 min at 5000 g and 10°C and then loaded with 11 mL of the mAb solution. The sample was centrifuged for 30 min at 10°C and 5000 g and the retained protein transferred to a 500 mL volumetric flask, for dilution using reaction buffer. The retained sample had a light-yellow color and was very viscous. In order to ensure complete recovery of the sample, aliquots of the buffer were periodically added to the filter to facilitate pipetting (using LoBind tips from Eppendorf).

**SDS Page:** was performed according to the BioRad TGX Precast Gels® specifications. 15 μL of rIgG sample were mixed with 5.0 μL of 4× Laemmli loading buffer from BioRad, heated at 90 °C for 5 min, cooled down and then loaded into the precast 4–15% Mini-PROTEAN® TGX™ protein gels from BioRad. 10 μL of pre-stained SDS-PAGE Standards (Bio-Rad) were used as the molecular marker. The gels were run using a Mini-PROTEAN Tetra cell from BioRad connected to a power supply at a constant voltage of 200 V in a 1× solution of Tris/Glycine/SDS running buffer at room temperature for approximately 45 min (or until the dye front reached the end the gel). Gels were fixed by microwaving for 50 seconds in a solution of 50% ethanol in water with 10% acetic acid and stained with Coomassie blue solution.([Merril, 1990](#_ENREF_8)) The gel was de-stained overnight in a solution of 50% methanol in water with 10% acetic acid and then scanned.

**Purification Procedure:** For simple purification, 500µL of the reaction mixture (the Alk4-IgG sample) was transferred to the Amicon ultracentrifuge filters, spun at 14,000 g for 5 minutes and then the filter was reloaded with a 450 µL (because of the low capacity of the centrifugal filters) aliquot of sample and at a time until all the sample had been passed through the filter. The retained sample was washed with the reaction buffer (with spinning at 14,000 g for 5 minutes) until there was no free linker being washed through (~8 × 400 µL washes), which was confirmed using absorbance spectroscopy. The purified sample was collected by reversing the filter and spinning (1,000 g for 2 minutes) and diluted with the reaction buffer to an approximate concentration of 1 g/L.

**Buffers for Blank subtraction:** The blanks used for blank subtraction in absorbance and fluorescence data were:

- PBS with EDTA for the starting material and purified product.
- PBS with EDTA and TCEP for the reduced intermediates.
- PBS/EDTA+TCEP+Linker for conjugates.
- PBS/EDTA+TCEP+Linker+Quencher for conjugates after quenching.

Concentrations of species are those used in the reactions (see main body text).

**Measurement of DAR by absorbance spectroscopy:** For the DAR calculation, the molar extinction coefficients at 248 and 280 nm of the antibody were obtained from the literature ([Hamblett et al., 2004](#_ENREF_3)) while those for the linker were calculated using solutions of known concentration of the linker. The following equation was used:

$DAR= \frac{\varepsilon_{248}^{IgG}-F{\times\varepsilon}_{280}^{\mathrm{IgG}}}{F{\times\varepsilon}_{280}^{Lnk}-\varepsilon_{248}^{\mathrm{Lnk}}}$

where F is the ratio between absorbance at 248 and 280nm and 280 ε*^Lnk^* and ε*^IgG^* are the molar concentrations of the linker and IgG respectively. ([Chen, 2013](#_ENREF_1); [Hamblett et al., 2004](#_ENREF_3)).

**Table S2:**  Summary description of sample types generated, data acquired, and the datasets used for chemometric analysis. In the main manuscript the datasets used for modelling are referenced according to the sample abbreviation, some of these are single sample types (e.g., Pur-ADC for DAR quantification by UV-visible) or are datasets constructed using multiple sample types (e.g., IgG-SM +Pur-ADC, Figure 2B-C). The specific spectral data being used for each model is detailed in the text.

| **Abbreviation** | **Description** | **Data Acquired** |
| --- | --- | --- |
| IgG-SM | IgG (mAb) in PBS buffer with EDTA | pEEM, UV, DLS, SEC, SDS-PAGE |
| Red1-IgG | Unpurified intermediate reduction reaction mixture, Sample contains: IgG, PBS/EDTA, TCEP.HCl  Measurement finished 10 minutes after TCEP was added. | pEEM, UV |
| Red2-IgG | Unpurified intermediate reduction reaction mixture, contains: IgG, PBS/EDTA, TCEP.HCl  Measurement finished 55 minutes after TCEP was added. | pEEM, UV |
| Red3-IgG | Final reduction reaction mixture, Sample contains: IgG, PBS/EDTA, TCEP.HCl  Measurements finished 120 minutes after TCEP was added. | pEEM, UV, DLS |
| Alkylation started by addition of excess drug linker to cuvette. | | |
| Alk1-IgG | Unpurified reaction mixture contains: IgG, PBS/EDTA, TCEP.HCl, Linker/DMSO. Measurement finished 10 minutes after linker was added. | pEEM, UV |
| Alk2-IgG | Unpurified reaction mixture contains: IgG, PBS/EDTA, TCEP.HCl, Linker/DMSO.  Measurement finished 55 minutes after linker was added. | pEEM, UV |
| Alk3-IgG | Unpurified reaction mixture contains: IgG, PBS/EDTA, TCEP.HCl, Linker/DMSO.  Measurement finished 100 minutes after linker was added. | pEEM, UV |
| Alkylation stopped after 2hours by addition of excess NAC. | | |
| Alk4-IgG | Unpurified final reaction mixture contains: IgG, PBS/EDTA, TCEP.HCl, Linker/DMSO, NAC. Measurement finished 10 minutes after NAC was added. | pEEM, UV, DLS |
| Pur-ADC | Final reaction mixture semi-purified by column to remove TCEP/NAC and unreacted linker/. | pEEM, UV, DLS, SEC, SDS-Page |

# Results and discussion:


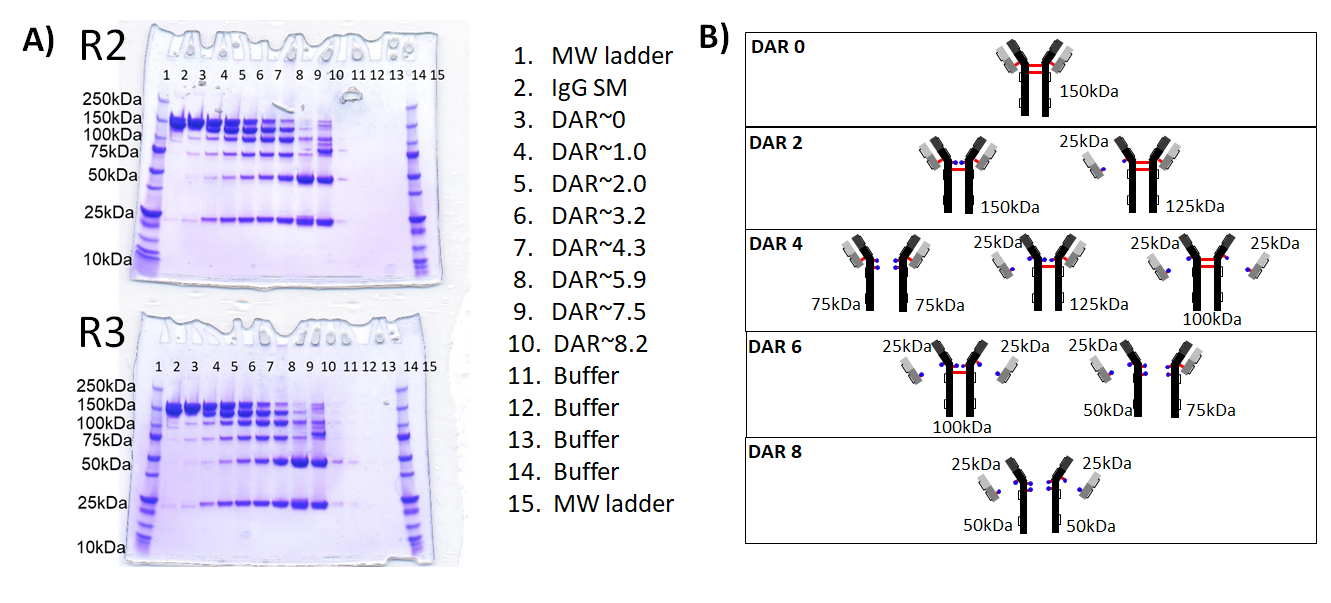


**Figure S4:** (A) SDS-PAGE gels with Coomassie blue staining of replicates 2 and 3 of purified ADC samples and controls. (B) Schematic diagram showing possible fragments generated by the conjugated mAb after contact with SDS.

**Table S3:** Selected DLS data including R_h_ (from main DLS peak of the distribution fit) and Z-average (radius) values for IgG-SM, reaction intermediates, and Pur-ADC samples. The Z-average values for the Red3-IgG and Alk4-IgG samples are not accurate size values as the PdI values of these measurements were all >0.5 and thus unsuitable for Cumulants fitting. They are included here to show the relative size changes being encountered during synthesis. The bottom part of the table shows the PdI values (from triplicate measurements) and the Derived count rate (this is the estimated count rate corrected for use of different attenuating filters…it is a measure of the actual scattered light intensity).

| **DAR** | **R_h_ (nm)** | | | | **Z-average (nm)** | | | |
| --- | --- | --- | --- | --- | --- | --- | --- | --- |
|  | **IgG-SM** | **Red3-IgG** | **Alk4-IgG** | **Pur-ADC** | **IgG-SM** | **Red3-IgG** | **Alk4-IgG** | **Pur-ADC** |
| **0** | 6.3 ± 0.2 | 6.1 ± 0.0 | 6.6 ± 0.1 | 8.2 ± 0.1 | 6.0 ± 1.0 | 10.5 ± 5.9 | 21.5 ± 11.8 | 7.5 ± 0.1 |
| **~1.0** | 6.4 ± 0.4 | 5.9 ± 0.1 | 6.6 ± 0.1 | 6.7 ± 0.1 | 5.8 ± 0.1 | 30.8 ± 19.1 | 37.1 ± 17.4 | 6.1 ± 0.1 |
| **~2.0** | 6.4 ± 0.2 | 6.1 ± 0.2 | 6.7 ± 0.1 | 7.5 ± 0.4 | 5.9 ± 0.1 | 11.2 ± 9.3 | 20.6 ± 12.3 | 8.8 ± 6.1 |
| **~3.2** | 6.4 ± 0.1 | 6.1 ± 0.1 | 6.7 ± 0.1 | 8.1 ± 0.4 | 5.8 ± 0.1 | 9.5 ± 1.1 | 28.3 ± 28.5 | 7.5 ± 0.1 |
| **~4.3** | 6.3 ± 0.1 | 6.1 ± 0.1 | 6.7 ± 0.1 | 7.6 ± 0.3 | 5.8 ± 0.1 | 12.7 ± 7.0 | 31.3 ± 31.1 | 8.2 ± 4.7 |
| **~5.9** | 6.4 ± 0.1 | 6.1 ± 0.1 | 6.7 ± 0.4 | 19.4 ± 1.6 | 5.9 ± 0.2^$^ | 11.6 ± 2.0 | 35.5 ± 27.7 | 13.1 ± 0.4 |
| **~7.5** | 6.7 ± 0.4 | 6.0 ± 0.0 | 6.6 ± 0.1 | 8.6 ± 0.1 | 5.8 ± 0.0 | 10.5 ± 1.6 | 25.4 ± 13.9 | 7.9 ± 0.2 |
| **~8.2** | 6.2 ± 0.1 | 6.1 ± 0.1 | 6.7 ± 0.3 | 9.5 ± 0.3 | 5.8 ± 0.2 | 9.2 ± 1.0 | 26.0 ± 6.9 | 8.55 ± 0.2 |
| **DAR** | **PdI (n=3)** | | | | **Derived Count Rate (kcps)*** | | | |
|  | **IgG-SM** | **Red3-IgG** | **Alk4-IgG** | **Pur-ADC** | **IgG-SM** | **Red3-IgG** | **Alk4-IgG** | **Pur-ADC** |
| **0** | 0.08 ± 0.01 | 0.53 ± 0.12 | 0.57 ± 0.21 | 0.22 ± 0.005 | 720 | 1112 | 1217 | 1033 |
| **~1.0** | 0.08 ± 0.02 | 0.51 ± 0.07 | 0.76 ± 0.12 | 0.15 ± 0.014 | 734 | 1358 | 1576 | 767 |
| **~2.0** | 0.08 ± 0.01 | 0.48 ± 0.03 | 0.68 ± 0.03 | 0.21 ± 0.014 | 731 | 1058 | 1325 | 984 |
| **~3.2** | 0.08 ± 0.01 | 0.55 ± 0.04 | 0.79 ± 0.04 | 0.22 ± 0.006 | 721 | 1088 | 1520 | 1061 |
| **~4.3** | 0.07 ± 0.01 | 0.54 ± 0.15 | 0.76 ± 0.08 | 0.18 ± 0.01 | 741 | 1200 | 1599 | 949 |
| **~5.9** | 0.12± 0.06 | 0.52 ± 0.09 | 0.63 ± 0.08 | 0.27 ± 0.008 | 747^$^ | 1168 | 1410 | 2377 |
| **~7.5** | 0.08 ± 0.01 | 0.51 ± 0.02 | 0.66 ± 0.07 | 0.21 ± 0.02 | 749 | 1119 | 1429 | 1085 |
| **~8.2** | 0.07 ± 0.01 | 0.51 ± 0.04 | 0.76 ± 0.17 | 0.24 ± 0.015 | 746 | 1080 | 1641 | 1289 |

*Average of three measurements. ^$^ Average of two measurements


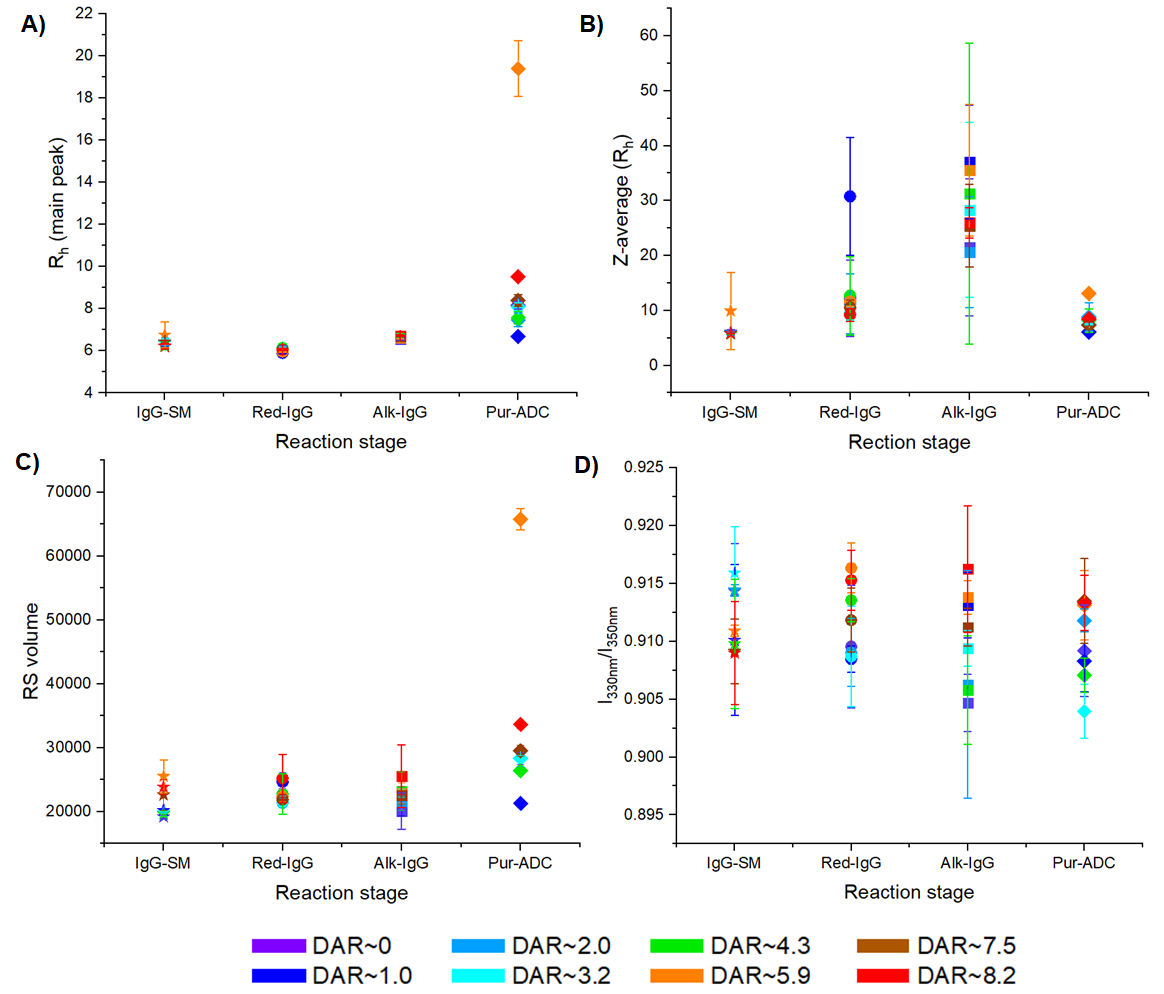


**Figure S5:**  (A) Hydrodynamic radius calculated from main peak of DLS data (distribution fit); (B) Z-average (radius, nm) values; (C) RS volume; and (D) ratio between two populations of Tryptophan (I_350_/I_330nm_ or more exposed to more buried).


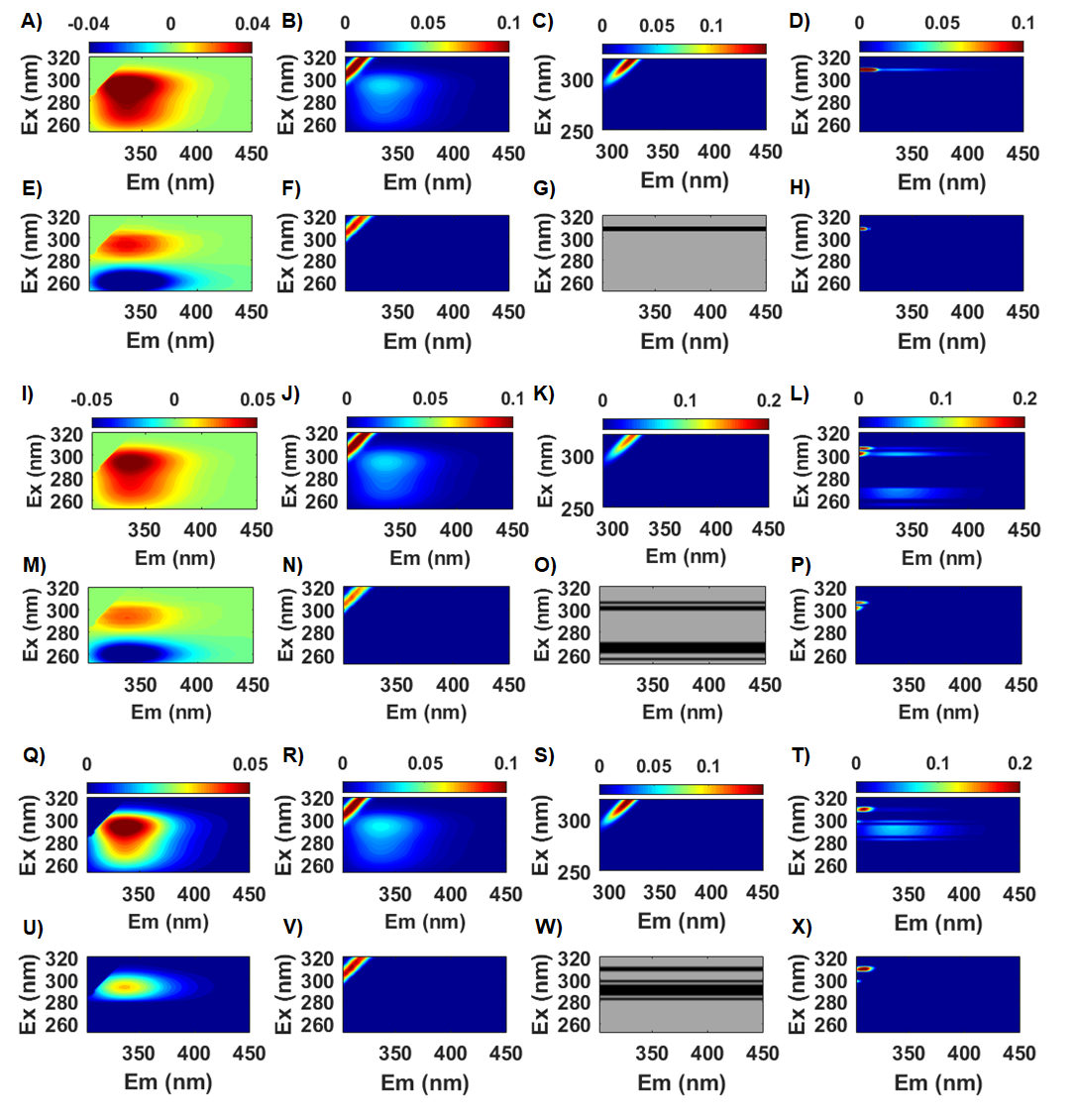


**Figure S6:** Loadings plots of Latent Variables (LV) obtained from u-PLS prediction models of: R_h_ (A-H) and %Agg. (I- P) of IgG-SM +Pur-ADC (n=48), and R_h_ (Q-X) of SM+Red3+Alk4+Pur-ADC (n=96). Quantification was carried out using the fluorescence signal from EEM_||_ (A/E, I/M, Q/U), the combined EEM_||_ + RS_||_ signal (B/F, J/N, R/V) and RS_||_ (C, K, S). G, O and W are the iPLS selected variables and D/H, L/P, and T/X are the LVs of prediction of R_h_, %Agg. of IgG-SM +Pur-ADC and R_h_ of IgG-SM +Red3+Alk4+Pur-ADC respectively using EEM_||_ + RS_||_ data after iPLS variable selection.

**ROBPCA Scores Analysis for reaction monitoring:**

PC1 of models of the IgG-SM + Red-IgG samples (max. at λ_ex/em_~276/342 nm) suggested a significant difference in scores of some starting materials (Figure **S7**, DAR~0, 5.9, 7.5, and 8.2), which was carried through the reduction reaction, although all reactions followed the same trajectory with the biggest variations in scores values occurring from IgG-SM to Red1-IgG. This might be related to the COVID19 induced time delay between reactions leading to high DAR (carried out in March 2020) and the other ones (carried out in November 2020), which were also carried out by different analysts. This was the same trend highlighted by ROBPCA of IgG-SM samples only, which separated two main groups of samples by PC1 (73.29%). While the IgG-SM samples used in March had positive scores, those used in November showed negative scores. However, the fact that IgG-SM used for control reactions were mis-grouped (these reactions were carried out in November but were grouped with the March samples), might suggest that instrument related variation might not be the only factor responsible for the clear discrimination of IgG-SM. PC2 explained a significant percentage of variance amongst IgG-SM (18.93%) and highlighted inter-replicate variation. Because the separation along PC2 is associated with changes in Trp emission when in different local environments, we can suggest that this inter-replicate variation might be caused by conformational changes that possibly changes the fluorophores local environment (Figure S7C). However, we estimate that the spectra of all starting materials changes by approximately 1.8% (EEM_RSD_), thus, this discrimination is based on very small variations in the spectra, which could also be impacted by instrument/measurement related changes.

PC2 of the IgG-SM +Red-IgG samples did only indicate intermediates of reactions leading to DAR~5.9 as outliers, which could be related with physical stability because these samples where the most stressed after purification. However, the variance explained by this PC was very low (2.87%) and so was the variance of IgG-SM and Red-IgG. For the models of the alkylation intermediates, the % of variance explained by PC2 was significantly bigger (20%) and highlighted changes in Trp environment. The scores shows a clear relationship with the final DAR, with reactions leading to low DAR having a much bigger change after the addition of the quencher. Because the score values are proportional to the DAR value and to the change in scores after the reaction was stopped, this could be related to the amount of free linker in solution which will be higher for low DAR conjugates and low for high DAR conjugates. After the reaction is stopped any free linker will have reacted with the quencher causing the scores of all reactions to decrease to similar values.

When the scores values were normalized to the first data point of each group, IgG-SM and Alk1-IgG respectively (**Figure S8**), it minimized the effect of starting material variance and emphasized reaction induced changes.


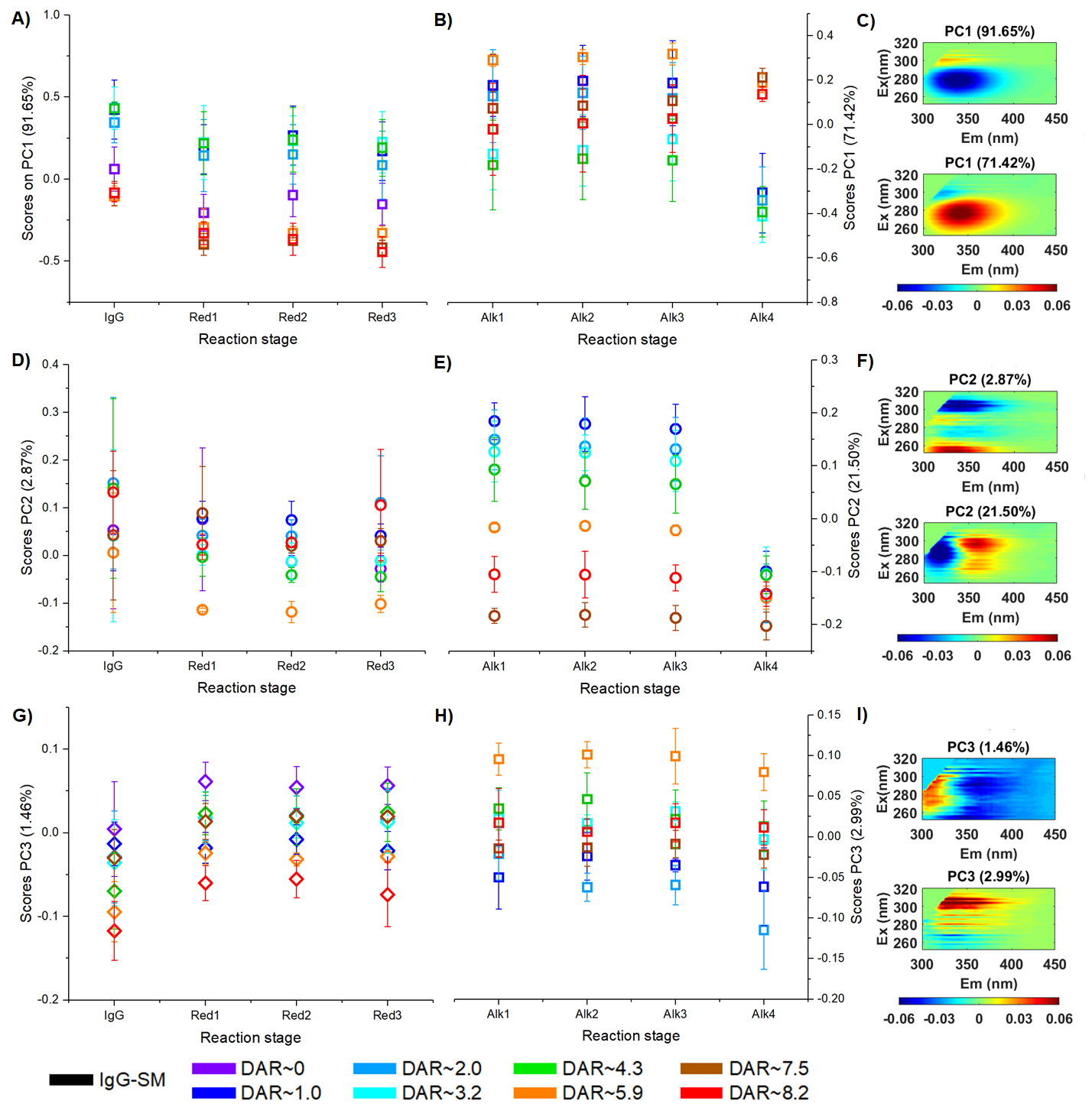


**Figure S7:**  ROBPC 1 (top row), 2 (middle row) and 3 (bottom row) scores from models including IgG + Red-IgG (A,D,G) and Alk-IgG (D,E,H). The colors indicate the different DAR of the final products (from 0-8). The results are shown as the mean±StdDev of triplicate reactions at each data point. (C/F/I) ) Refolded loadings plot of ROBPC1,2 and 3 respectively obtained from models of IgG + Red-IgG (top) and Alk-IgG (bottom). The data used were the (EEM**_||_** spectra.


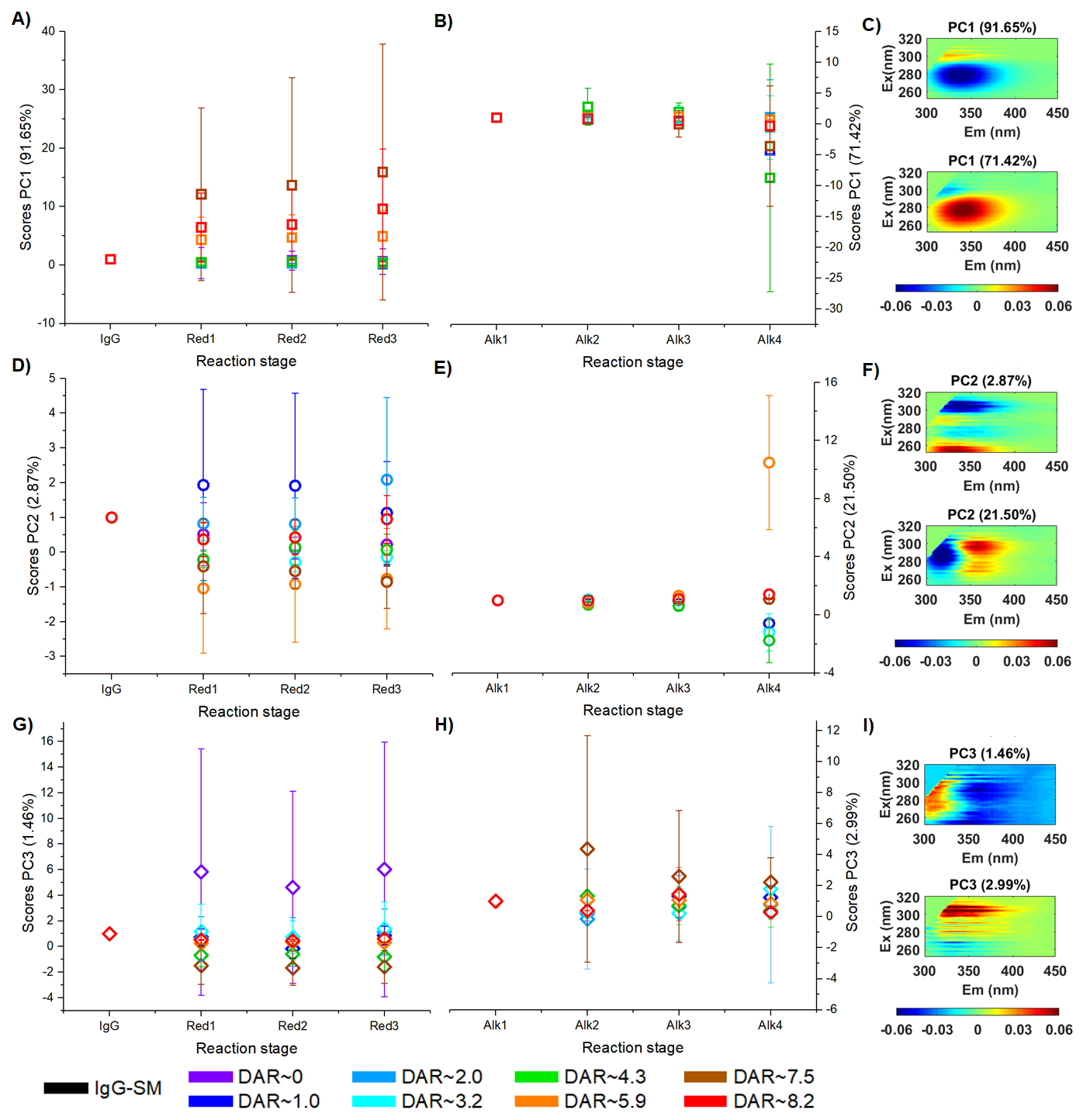


**Figure S8:**  ROBPC 1 (top row), 2 (middle row) and 3 (bottom row) scores from models including IgG + Red-IgG (A,D,G) and Alk-IgG (D,E,H). The results are shown as the mean±StdDev of triplicate reactions at each data point and values were normalized to scores of each IgG-SM or Alk1-IgG . The colors indicate the different DAR of the final products (from 0-8). Refolded loadings plot of ROBPC1,2 and 3 (C,F and I, respectively) obtained from models of IgG + Red-IgG (top) and Alk-IgG (bottom).

**Table S4:**  Results of sensitivity (TPR), Specificity (TNR) and misclassification error (Err) in % for SVM classification models of UV-Visible, EEM_ǁ_, EEM_⊥_, and EEM_T_ measurements according to the reaction step.

| **Calibration results** | | | | | | | | | | |
| --- | --- | --- | --- | --- | --- | --- | --- | --- | --- | --- |
| **Actual** | **IgG** | **Red Low** | **Red Mid** | **Red High** | **Alk Low** | **Alk Mid** | **Alk**  **High** | **Pur Low** | **Pur Mid** | **Pur High** |
| **Sensitivity** | 94 | 100 | 100 | 100 | 100 | 100 | 100 | 100 | 100 | 100 |
| **Specificity** | 100 | 100 | 99 | 100 | 100 | 100 | 100 | 100 | 100 | 100 |
| **Error** | 7 | 0 | 7 | 0 | 0 | 0 | 0 | 0 | 0 | 0 |
| **CV results** | | | | | | | | | | |
| **Actual** | **IgG** | **Red Low** | **Red Mid** | **Red High** | **Alk Low** | **Alk Mid** | **Alk**  **High** | **Pur Low** | **Pur Mid** | **Pur High** |
| **Sensitivity** | 67 | 93 | 100 | 95 | 94 | 100 | 100 | 100 | 100 | 100 |
| **Specificity** | 98 | 98 | 98 | 99 | 100 | 99 | 100 | 100 | 100 | 100 |
| **Error** | 6 | 3 | 1 | 1 | 1 | 1 | 0 | 0 | 0 | 0 |
| **Validation results** | | | | | | | | | | |
| **Actual** | **IgG** | **Red Low** | **Red Mid** | **Red High** | **Alk Low** | **Alk Mid** | **Alk**  **High** | **Pur Low** | **Pur Mid** | **Pur High** |
| **Sensitivity** | 50 | 75 | 100 | 100 | 83 | 100 | 100 | 50 | 100 | 100 |
| **Specificity** | 98 | 98 | 93 | 100 | 100 | 100 | 100 | 100 | 100 | 100 |
| **Error** | 8 | 4 | 6 | 0 | 0 | 0 | 0 | 2 | 0 | 0 |

Sensitivity=TPR (True positive rate), Specificity=TNR (True negative rate)

**Table S5:**  UV-AI values for the seven reaction timepoints from all eight reactions (done in triplicate).

| **DAR** | 0 | | | 1 | | | 2 | | | 3.2 | | |
| --- | --- | --- | --- | --- | --- | --- | --- | --- | --- | --- | --- | --- |
| TCEP conc. | 0 | | | 1.25 | | | 2.5 | | | 5 | | |
| IgG-SM | 0.77 | 0.40 | 0.30 | 0.40 | 0.45 | 0.39 | 0.42 | 0.43 | 0.29 | 0.43 | 0.33 | 0.26 |
| Red-IgG | 2.14 | 0.67 | 0.72 | 2.26 | 2.04 | 2.12 | 1.26 | 0.71 | 0.56 | 0.84 | 1.21 | 0.58 |
| Alk1 | 2.32 | 0.73 | 0.68 | 3.08 | 2.82 | 2.89 | 2.14 | 1.50 | 1.46 | 1.60 | 1.91 | 1.38 |
| Alk2 | 2.58 | 0.70 | 0.67 | 3.30 | 2.97 | 3.06 | 2.38 | 1.62 | 1.72 | 1.89 | 2.23 | 1.53 |
| Alk3 | 2.67 | 1.29 | 0.75 | 3.54 | 3.17 | 3.27 | 2.62 | 1.76 | 1.88 | 2.15 | 2.55 | 1.78 |
| Alk-IgG | 2.72 | 0.75 | 0.81 | 2.71 | 2.61 | 2.52 | 1.96 | 1.21 | 1.49 | 1.72 | 2.11 | 1.44 |
| Pur-ADC | 0.63 | 0.88 | 0.59 | 0.69 | 0.61 | 0.95 | 0.75 | 0.68 | 0.70 | 0.71 | 0.64 | 0.79 |
| **DAR** | 4.3 | | | 5.9 | | | 7.5 | | | 8.2 | | |
| TCEP conc. | 7.5 | | | 10 | | | 25 | | | 50 | | |
| IgG-SM | 0.67 | 0.36 | 0.31 | 1.02 | 1.45 | 0.35 | 1.89 | 1.21 | 0.39 | 1.10 | 0.78 | 0.33 |
| Red-IgG | 1.35 | 0.55 | 0.83 | 1.98 | 1.58 | 1.66 | 2.15 | 1.08 | 1.48 | 1.85 | 1.13 | 1.56 |
| Alk1 | 1.89 | 1.10 | 1.47 | 2.56 | 2.03 | 2.10 | 2.13 | 1.20 | 1.60 | 1.87 | 1.33 | 1.68 |
| Alk2 | 2.35 | 1.21 | 1.77 | 3.42 | 2.19 | 2.60 | 2.56 | 1.38 | 1.91 | 2.52 | 1.55 | 1.84 |
| Alk3 | 2.87 | 1.37 | 2.09 | 4.15 | 2.48 | 2.59 | 2.99 | 1.63 | 2.20 | 2.64 | 1.86 | 2.09 |
| Alk-IgG | 2.86 | 1.00 | 1.73 | 3.29 | 2.28 | 2.39 | 3.35 | 1.81 | 2.49 | 3.45 | 2.17 | 2.18 |
| Pur-ADC | 0.67 | 0.62 | 0.60 | 0.78 | 0.67 | 0.55 | 0.80 | 0.42 | 0.70 | 1.17 | 0.77 | 0.77 |


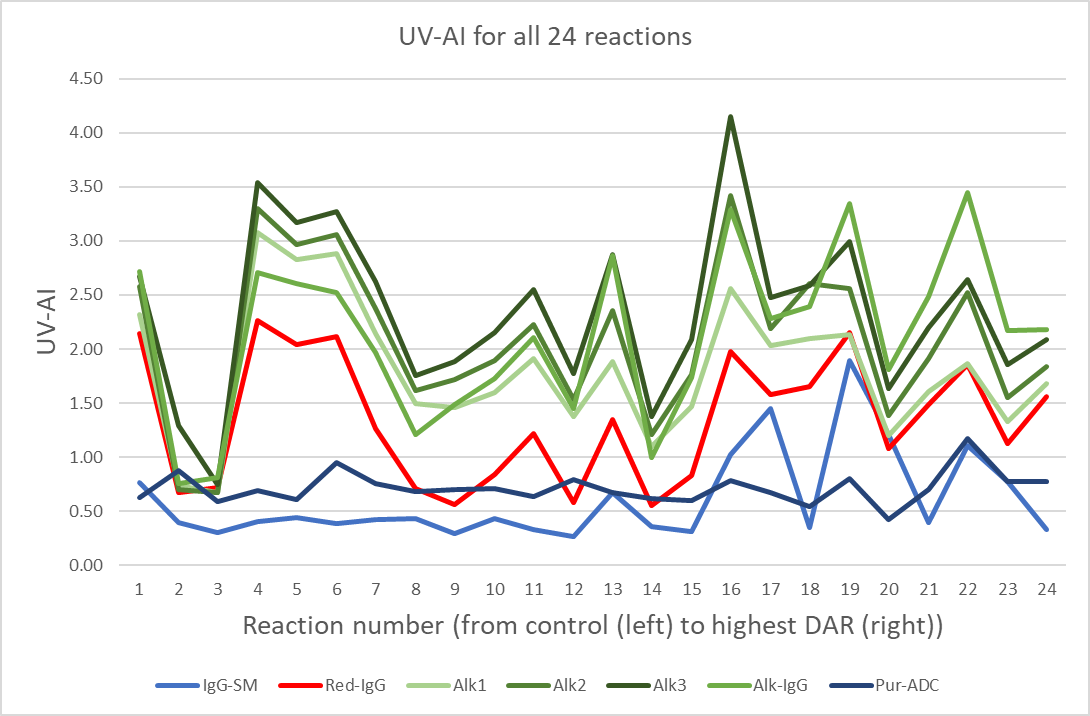


**Figure S9:**  Plot of UV-AI values against reaction number for all 8 reactions (done in triplicate) showing the differences between the different intermediates.

# References

Chen, Y. (2013). Drug-to-Antibody Ratio (DAR) by UV/Vis Spectroscopy. *Antibody-Drug Conjugates, 1045*, 267-273. doi:10.1007/978-1-62703-541-5_16

Ellman, G. L. (1959). Tissue sulfhydryl groups. *Arch Biochem Biophys, 82*(1), 70-77. doi:10.1016/0003-9861(59)90090-6

Hamblett, K. J., Senter, P. D., Chace, D. F., Sun, M. M. C., Lenox, J., Cerveny, C. G., . . . Francisco, J. A. (2004). Effects of drug loading on the antitumor activity of a monoclonal antibody drug conjugate. *Clinical Cancer Research, 10*(20), 7063-7070. doi:Doi 10.1158/1078-0432.Ccr-04-0789

Han, J. C., & Han, G. Y. (1994). A procedure for quantitative determination of tris(2-carboxyethyl)phosphine, an odorless reducing agent more stable and effective than dithiothreitol. *Analytical Biochemistry, 220*(1), 5-10. doi:10.1006/abio.1994.1290

Hermanson, G. T. (2008). *Bioconjugate techniques* (2nd ed.). Amsterdam ; Boston: Elsevier Academic Press.

ISO, IEC, OIML, & BIPM. (1995). GUIDE 98-1 Guide to the Expression of Uncertainty in Measurement. In. Geneva, Switzerland v.122.

Liu, H. C., & May, K. (2012). Disulfide bond structures of IgG molecules Structural variations, chemical modifications and possible impacts to stability and biological function. *Mabs, 4*(1), 17-23. doi:10.4161/mabs.4.1.18347

Merril, C. R. (1990). [36] Gel-staining techniques. In M. P. Deutscher (Ed.), *Methods in Enzymology* (Vol. 182, pp. 477-488): Academic Press.
